# Breaking aromaticity in the anaerobic degradation pathway of phenanthrene comprises only ATP-independent type III aryl-CoA reductases

**DOI:** 10.1038/s42003-026-10639-5

**Published:** 2026-07-13

**Authors:** Nadia A. Samak, Marvin Häßler, Oliver J. Schmitz, Khadija Adjir, Rainer U. Meckenstock

**Affiliations:** 1 Environmental Microbiology and Biotechnology (EMB), Faculty of Chemistry, University of Duisburg-Essen, Universitätsstr. 5, Essen, Germany; 2 Applied Analytical Chemistry, Faculty of Chemistry, University of Duisburg-Essen, Universitätsstr. 5, Essen, Germany; 3 Laboratory of Thermodynamics and Molecular Modeling, Faculty of Chemistry, University of Sciences and Technology Houari Boumediene (USTHB), BP32 El Alia, Bab Ezzouar, Algeria

**Keywords:** Environmental biotechnology, Environmental microbiology

## Abstract

The anaerobic degradation pathway of phenanthrene starts with activation through carboxylation to 2-phenanthroic acid, followed by thioesterification to produce 2-phenanthroyl-CoA, which is then reduced to hexahydro-2-phenanthroyl-CoA by two ATP-independent type III aryl-CoA reductases (AprB and AprC) to overcome the resonance energy of the aromatic rings. In this study, we elucidated the reduction of hexahydro-2-phenanthroyl-CoA to octahydro-2-phenanthroyl-CoA and its subsequent reduction to the fully dearomatized diene decahydro-2-phenanthroyl-CoA. The two-electron reduction steps were catalyzed by the heterologously produced and purified hexahydro-2-phenanthroyl-CoA reductase (AprD) and octahydro-2-phenanthroyl-CoA reductase (AprE). AprD and AprE have specific activities of 12.7 and 6.3 nmol min^-1^ mg^-1^ and *K*_M_ values of 15.1 and 63.9 nM, respectively. The ATP-independent and oxygen-sensitive reduction of hexahydro-2-phenanthroyl-CoA and octahydro-2-phenanthroyl-CoA catalyzed by AprD and AprE preferred dithionite-reduced methyl viologen as in vitro electron donor. AprD and AprE are monomeric enzymes with a molecular mass of ≈ 73 kDa and contain one FMN, one FAD, and one 4Fe-4S cluster, indicating that both reductases belong to the new type III aryl-CoA reductases of the old-yellow enzyme (OYE) family. The de-aromatization of 2-phenanthroyl-CoA aromatic rings through reduction was followed by water addition at the β-position of the cyclic diene, decahydro-2-phenanthroyl-CoA, producing β-hydroxydodecahydro-2-phenanthroyl-CoA. The hydration reaction was catalyzed by a homo-dimeric enoyl-CoA hydratase (ApcA) with a native molecular mass of ≈ 60 kDa and subunit molecular mass of ≈ 29 kDa. ApcA has a specific activity of 3.2 nmol min^-1^ mg^-1^ and a *K*_M_ value of 31.5 nM at pH value of 7.5. The catalytic activity of ApcA was oxygen-insensitive and the reaction did not need any cofactor or metal ion. These findings reveal a novel strategy in the anaerobic degradation of aromatic hydrocarbons where only ATP-independent type III aryl-CoA reductases are involved in breaking the aromaticity of the ring system before the metabolites enter subsequent beta-oxidation reactions.

## Abstract (web summary)

After phenanthrene activation to a CoA-thioester, the aromatic ring systems are fully reduced by type III aryl-CoA reductases without additional energy input through ATP-hydrolysis. This constitutes a new anaerobic degradation strategy that is distinct from the degradation of naphthalene

## Introduction

Polycyclic aromatic hydrocarbons (PAHs) are widespread in the environment and exhibit negative health and environmental impacts due to their toxic, mutagenic, and carcinogenic effects^1,2^. Only microorganisms can completely degrade PAHs to carbon dioxide^3^. Whereas aerobic microorganisms perform an oxidative strategy to activate the chemically stable PAHs and to break the aromaticity with the help of oxygenases and the very reactive elemental oxygen, anaerobic microorganisms activate non-substituted PAHs by carboxylation and, then, overcome the aromatic resonance energy by reducing the aromatic rings^4^. There are three different strategies known to reduce aromatic rings in anaerobic degradation. The first strategy is catalyzed by ATP-dependent oxygen-sensitive type I aryl-CoA reductases that utilize ferredoxin as a natural electron donor^5^. The prototype is benzoyl-CoA reductase, which is commonly found in benzoyl-CoA degradation by facultative anaerobes, such as *Thauera aromatica*^6^, *Azoarcus* strain CIB^7^, or *Rhodopseudomonas palustris*^8^. The second strategy comprises ATP-independent oxygen-sensitive type II aryl-CoA reductases that also reduce benzoyl-CoA. These enzymes contain tungsten and were discovered in strict anaerobes, such as the iron-reducing bacterium *Geobacter metallireducens* and the sulfate-reducing bacterium *Desulfococcus multivorans*^9–14^. Type II aryl-CoA reductases utilize energy from electron bifurcation to reduce the benzene ring structure. The third type is the ATP-independent and oxygen-insensitive type III aryl-CoA reductases, which belong to the old-yellow enzyme (OYE) family and are only known for anaerobic degradation of PAHs and were first discovered in the sulfate-reducing Deltaproteobacteria N47 and strain NaphS2^15–19^. Enzymes belonging to OYE are characterized by their yellowish color due to the presence of flavin cofactors, such as flavin mononucleotide (FMN) and flavin adenine dinucleotide (FAD)^20^. Although type III aryl-CoA reductases belong to the OYE family, not all OYE enzymes are type III aryl-CoA reductases. OYE has more functions, such as catalyzing the NADPH-linked reduction of nitro-olefins^21^, and the stereoselective reduction of a wide variety of small molecules, including xenobiotic toxins^22^. So far, only the anaerobic biodegradation pathways of naphthalene and methylnaphthalene have been partly elucidated, while knowledge of the anaerobic biodegradation of phenanthrene and larger-PAHs (**1**) is still scarce^16,18,23–25^.

In this study, we continue our investigation to uncover the upper anaerobic degradation pathway of phenanthrene (**1**) of the sulfate-reducing, phenanthrene-degrading enrichment culture TRIP 1, which was enriched from the Pitch Lake in La Brea, Trinidad, with phenanthrene (**1**) as the sole electron source^18,26,27^. The dominant organism (60.4%) of culture TRIP 1 belongs to the genus *Desulfatiglans* within the family *Desulfobacteraceae*^26,27^. Culture TRIP 1 also contained other microorganisms, such as *Paludibacter* (19.6%), *Desulfobulbaceae* (6.8%), *Spirochete* (3.4%), *Desulfomicrobium* (1.8%), and *Desulfarculus* (1.3%)^27^.

**Fig. 1:**
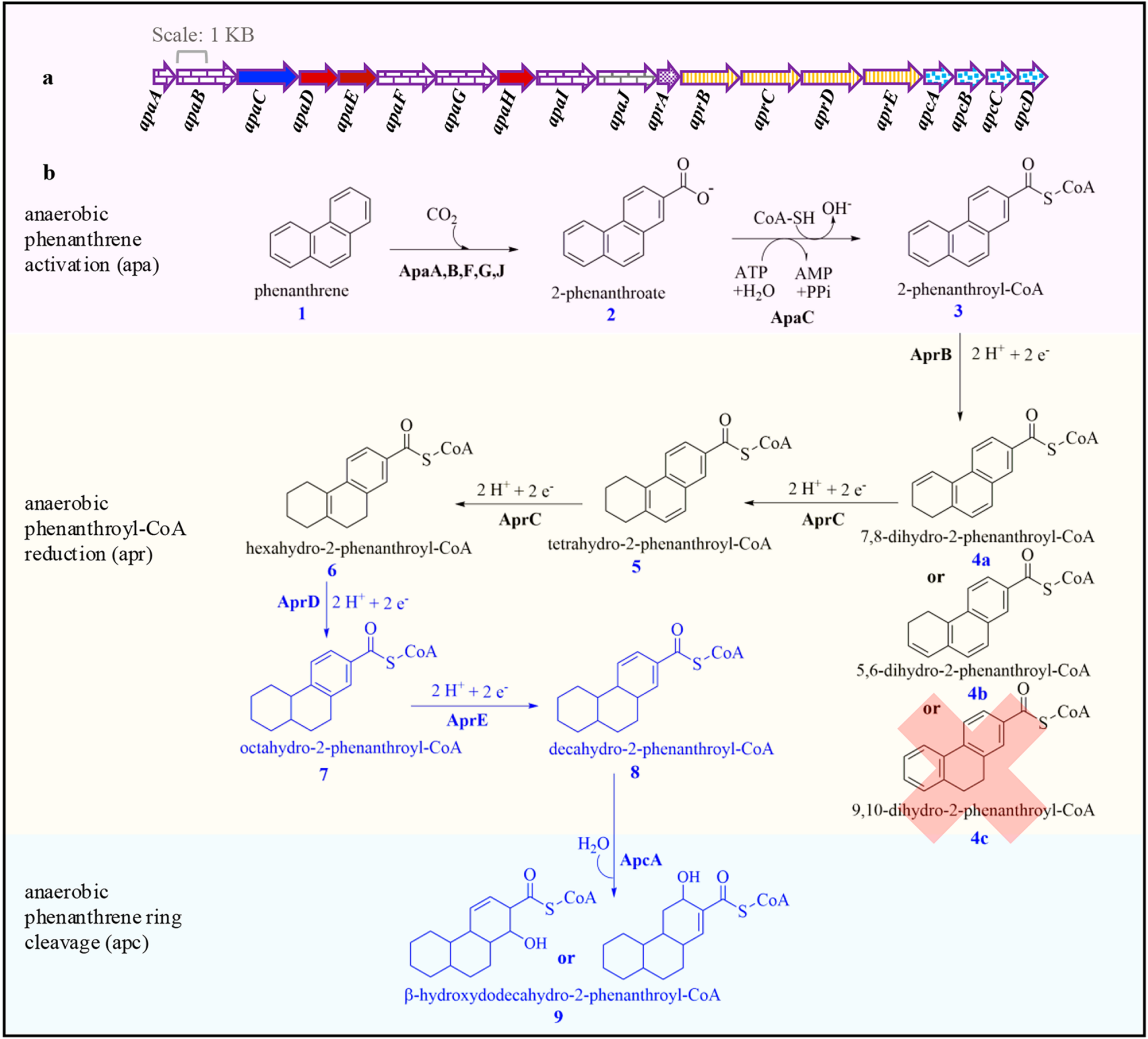
Anaerobic upper degradation pathway of phenanthrene. **a** Graphical presentation of the gene cluster encoding enzymes putatively responsible for anaerobic phenanthrene activation (*apaA-apaJ*)^25,26^, anaerobic phenanthrene ring reduction (*aprA-aprE*)^18,19^, including the hexahydro-2-phenanthroyl-CoA reductase (*aprD*, yellow vertical strips) and the octahydro-2-phenanthroyl-CoA reductase (*aprE*, yellow vertical strips), and anaerobic phenanthrene ring cleavage (*apcA-D*), starting with an enoyl-CoA hydratase (*apcA*, light blue large confetti). **b** proposed anaerobic upper degradation pathway of phenanthrene (**1**). Confirmed enzyme reactions are shown in black. Reactions analyzed in this study are marked in blue. The displayed isomers of the enzymatic products are hypothetical, since the precise locations of the saturated bonds are not determined yet. Compounds mentioned in this study were numbered between square brackets in the whole text and colored in blue in all figures. The red cross over compound (**4c**) indicates that this compound is not a real product of the reduction of 2-phenanthroyl-CoA catalyzed by AprB reductase^18^.

The initial steps of the anaerobic degradation pathway of phenanthrene (**1**), using enzymes encoded by the genes of the genus *Desulfatiglans* in enrichment culture TRIP 1, follow the same biochemical principles as the upper degradation pathway of the smaller two-ring PAH naphthalene^18,19,25,27^. Phenanthrene (**1**) is first activated by a UbiD-like carboxylase to produce 2-phenanthroic acid (**2**)^26^, followed by CoA-thioesterification catalyzed by 2-phenanthroate: CoA ligase (ApaC) to produce 2-phenanthroyl-CoA (**3**)^25^ (anaerobic phenanthrene activation (apa), Fig. 1a). The gene encoding 2-phenanthroate: CoA ligase (*apaC*)^25^ is located in the vicinity of the putative carboxylase gene cluster (*apaA,B,F,G, I*, and *J*)^27^ (Fig. 1a). Then, phenanthroyl-CoA is reduced by type III aryl-CoA reductases of the OYE family overcoming the resonance energy of the aromatic ring-system^18,19^ (anaerobic phenanthroyl-CoA reduction (apr), Fig. 1b). Four genes (*aprB-E*, Fig. 1a and Supplementary Fig. 1) encoding type III aryl-CoA reductases with 37–-57% amino acid sequence identities to 2-naphthoyl-CoA reductase and dihydro-2-naphthoyl-CoA reductase in *Deltaproteobacteria* N47 and strain NaphS2 were found in the *Desulfatiglans* metagenome of culture TRIP 1^27^. 2-Phenanthroyl-CoA (**3**) is reduced by the ATP-independent type III aryl-CoA reductase 2-phenanthroyl-CoA reductase (AprB) to produce two possible isomers of 7,8-dihydro-2-phenanthroyl-CoA (**4a**) or 5,6-dihydro-2-phenanthroyl-CoA (**4b**)^18^. The following reduction step is catalyzed by another ATP-independent type III reductase, namely dihydro-2-phenanthroyl-CoA reductase (AprC), which performs two consecutive two-electron reductions, converting the 7,8-dihydro-2-phenanthroyl-CoA (**4a**) or 5,6-dihydro-2-phenanthroyl-CoA (**4b**) to hexahydro-2-phenanthroyl-CoA (possibly 5,6,7,8,9,10-hexahydro-2-phenanthroyl-CoA) (**6**)^19^.

Up to this step, the upper anaerobic phenanthrene degradation pathway (**1**) is very similar to that of naphthalene by employing ATP-independent oxygen-insensitive type III aryl-CoA reductases (i.e., the reduction of 2-naphthoyl-CoA to 5,6-dihydro-2-naphthoyl-CoA and the subsequent reduction of the latter to 5,6,7,8-tetrahydro-2-naphthoyl-CoA in sulfate-reducing *Deltaproteobacteria* N47 and strain NaphS2)^15,17^. However, the next step in anaerobic naphthalene degradation is an ATP-dependent reduction of the remaining benzene ring of tetrahydro-2-naphthoyl-CoA to hexahydro-2-naphthoyl-CoA, which employs a type I aryl-CoA reductase similar to benzoyl-CoA reductases of the *Azoarcus*-type^7,16,28,29^. In strains N47 and NaphS2, the genes for this ATP-dependent type I reductase are located in direct vicinity to the two type III reductases^15,17,24,27^. In the metagenome of the anaerobic phenanthrene enrichment culture TRIP 1, genes sharing 40% nucleotide sequence similarity with type I reductases were found; however, these genes are not found in the neighborhood of the type III aryl-CoA reductase genes^18,19,27^, raising the question of how and to what extent the aromatic ring system of phenanthroyl-CoA gets reduced. This is a very important problem because, although the total ∆G^0’^ of reducing a benzene ring is exergonic and more or less in the same range for the different reduction steps of monoaromatic compounds and PAHs, the activation energies to overcome the resonance energy of a benzene ring are very high at standard conditions and pH 7 (>100 kJ mol^-1^)^30^. For type I aryl-CoA reductases reducing monoaromatic compounds or tetrahydro-2-naphthoyl-CoA, this problem is solved by the expense of ATP-hydrolysis. Hence, it is unclear how anaerobic microorganisms can use type III aryl-CoA reductases to reduce the aromatic ring systems of larger PAHs with three or more rings (e.g., phenanthrene) without additional energy input from ATP hydrolysis, rather than using type I aryl-CoA reductases.

Genes encoding the type III aryl-CoA reductases in the metagenome of the anaerobic, phenanthrene-degrading *Desulfatiglans* TRIP 1 are surrounded by putative hydratase, dehydrogenase, hydrolase, and thiolase encoding genes (*apcA-D*, anaerobic phenanthrene ring cleavage (apc), Fig. 1a), constituting a gene cluster with high nucleotide sequence identity to the tetrahydro-2-naphthoyl-CoA (*thn)* gene cluster of the naphthalene-degrading *Deltaproteobacterium* N47^27^. The products of the *thn* gene cluster catalyze β-oxidation-like reactions following the reduction steps in anaerobic naphthalene degradation^1^.

In this study, we elucidated how the aromatic ring system in anaerobic phenanthrene degradation is reduced to a non-aromatic metabolite, followed by a hydratase reaction, thereby demonstrating the continuous reaction sequence and the transition from ring reduction to beta-oxidation. Therefore, we heterologously expressed the remaining two genes of putative type III aryl-CoA reductases (*aprD* and *aprE*) as well as an enoyl-CoA hydratase gene (*apcA*) in *E. coli*. The gene products were purified and characterized, and their role in the anaerobic degradation of phenanthrene was investigated with enzyme assays. As a result, we elucidated the complete reduction pathway and the transition to beta-oxidation during anaerobic phenanthrene degradation.

## Results

### Biochemical properties of the overproduced oxidoreductases (AprD and AprE) and enoyl-CoA hydratase (ApcA)

**Fig. 2:**
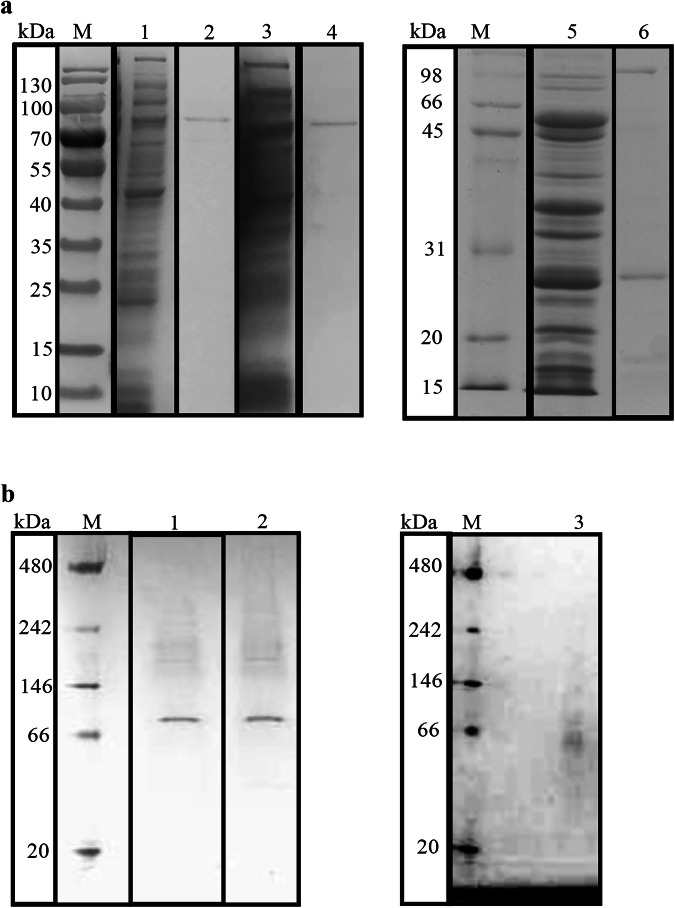
Purification of hexahydro-2-phenanthroyl-CoA reductase (AprD), octahydro-2-phenanthroyl-CoA reductase (AprE), and the enoyl-CoA hydratase (ApcA). **a** SDS-PAGE analysis of AprD, AprE, and ApcA before and after Strep-Tactin affinity column purification. M, protein MW standard; lane 1, *E. coli* cell-free extract with AprD overproduction, lane 2, purified AprD; lane 3, *E. coli* cell-free extract with AprE overproduction, lane 4, purified AprE; lane 5, *E. coli* cell-free extract with ApcA overproduction, lane 6, purified ApcA. The second band at 98 kDa in lane 6 is an impurity. **b** Blue native-PAGE analysis of purified AprD (lane 1), AprE (lane 2), and ApcA (lane 3).

The *aprD*, *aprE*, and *apcA* genes encoding the hexahydro-2-phenanthroyl-CoA reductase, octahydro-2-phenanthroyl-CoA reductase, and enoyl-CoA hydratase, respectively, were heterologously overproduced in *E. coli*. The soluble enzymes were overproduced aerobically in *E. coli* as C-terminal Twin-Strep-tag-fusion proteins. Approximately 3–3.5 mg of protein were produced from an *E. coli* wet mass of 10 g. SDS-PAGE analysis confirmed the purity of AprD and AprE, both showing pure enzymes with a molecular mass of ≈ 73 kDa (Fig. 2a). Enoyl-CoA hydratase (apcA) showed a molecular mass of ≈ 29 kDa, but an impurity band was observed at ≈ 100 kDa (Fig. 2a). Blue native gel electrophoresis confirmed the monomeric native state of the purified AprD and AprE proteins with a molecular mass of ≈ 73 kDa (Fig. 2b). Under native conditions, enoyl-CoA hydratase (ApcA) was found to form a homodimer, confirmed by a protein band with a molecular mass of ≈ 60 kDa (Fig. 2b).

**Fig. 3:**
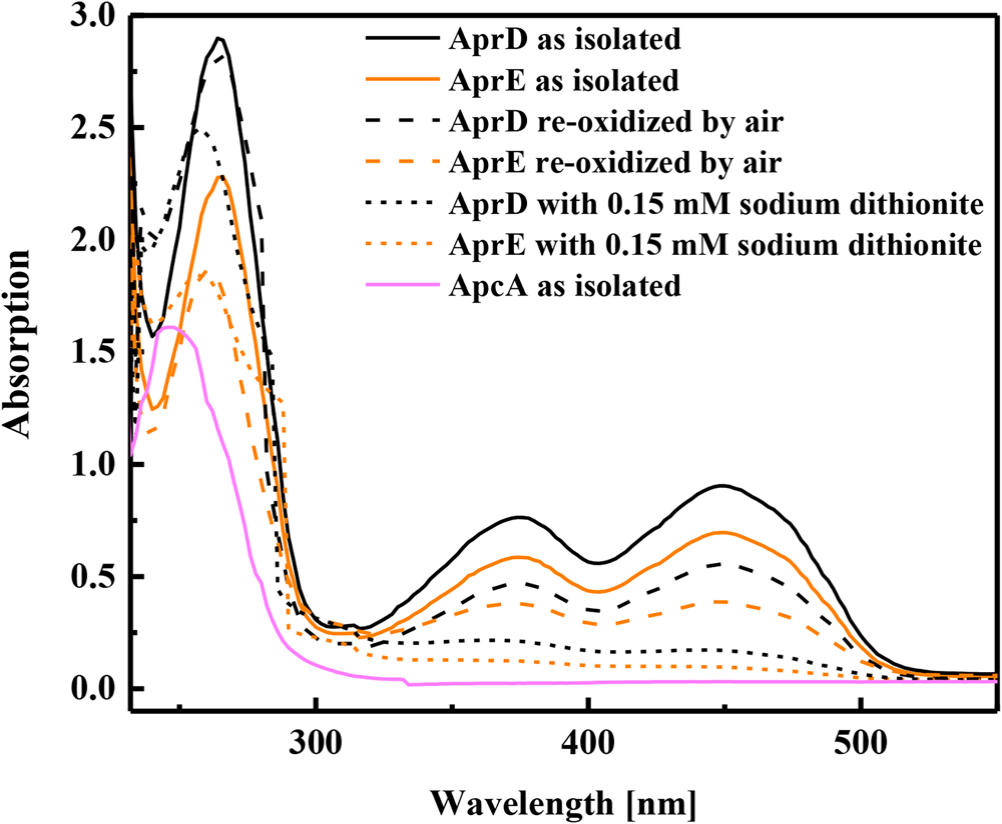
UV/vis spectra of hexahydro-2-phenanthroyl-CoA reductase (AprD, black color), octahydro-2-phenanthroyl-CoA reductase (AprE, orange color), and enoyl-CoA hydratase (ApcA, magenta color). Spectra were recorded for each protein (20 µM) as isolated and for AprD and AprE after flavin reduction by adding 0.15 mM sodium dithionite, and after flavin re-oxidation by exposing the enzymes to air.

Gene sequence analysis of *aprD* and *aprE* showed that both hexahydro-2-phenanthroyl-CoA reductase and octahydro-2-phenanthroyl-CoA reductase, encoded by these two genes, belong to the OYE family, which is characterized by the presence of flavin cofactors. The oxidized flavin cofactors of AprD and AprE (enzymes as isolated) showed a characteristic peak at 375 and 450 nm in the UV/vis spectrum (Fig. 3). The flavin cofactors of AprD and AprE were completely reduced after adding 0.15 mM sodium dithionite. Both enzymes were exposed to oxygen to re-oxidize the flavins which reestablished their characteristic peaks at 375 and 450 nm in the UV/vis spectra (Fig. 3). The flavin mononucleotide (FMN) content of both enzymes was quantified with liquid chromatography/mass spectrometry (LC/MS) and compared with commercial standards, giving an average FMN content of 0.7 and 0.6 per monomer of AprD and AprE, respectively. The flavin adenine dinucleotide (FAD) content of both enzymes was 0.9 and 0.7 per monomer of AprD and AprE, respectively. The iron content of AprD and AprE was 3.5 and 2.9 Fe/monomer, respectively, suggesting the presence of one 4Fe-4S cluster per monomer in both enzymes (Table 1). The UV/vis spectra of enoyl-CoA hydratase (ApcA) showed only an absorption peak at 280 nm, corresponding to aromatic amino acid residues of the enzyme and confirming that the enzyme did not contain flavin cofactors.

**Table 1:** Properties of the overproduced recombinant proteins

Parameter	AprD	AprE	ApcA
Specific activity (nmol min^-1^ mg^-1^) at *V*_max_	12.7 ± 4.1	6.3 ± 2.3	3.2 ± 0.9
Apparent *K*_M_ (nM)	15.1 ± 4.2	63.9 ± 9.3	31.5 ± 4.1
FMN content	0.7 ± 0.4 FMN/monomer	0.6 ± 0.5 FMN/monomer	^*^ND
FAD content	0.9 ± 0.6 FAD/monomer	0.7 ± 0.3 FAD/monomer	^*^ND
Iron content	3.5 ± 0.3 Fe/monomer	2.9 ± 0.5 Fe/monomer	^*^ND
^* *^Used electron donors	Dithionite-reduced methyl viologen: 100% ^***^NADH: 5% Dithionite: 0% Ti(III)-citrate: 0% NADPH: 0%	Dithionite-reduced methyl viologen: 100% NADH: 0% Dithionite: 0% Ti(III)-citrate: 0% NADPH: 0%	- - - - -
Enzymatic assay oxygen sensitivity	yes	yes	No

^*^ND not detected.

^**^Activity of other electron donors given in % of the maximal activity measured with reduced methyl viologen.

^***^ The activity obtained with NADH corresponded to approximately 5% of the maximal activity observed with dithionite-reduced methyl viologen as the electron donor, which was defined as 100% activity.

### ATP-independent reduction of hexahydro-2-phenanthroyl-CoA to decahydro-2-phenanthroyl-CoA

**Fig. 4:**
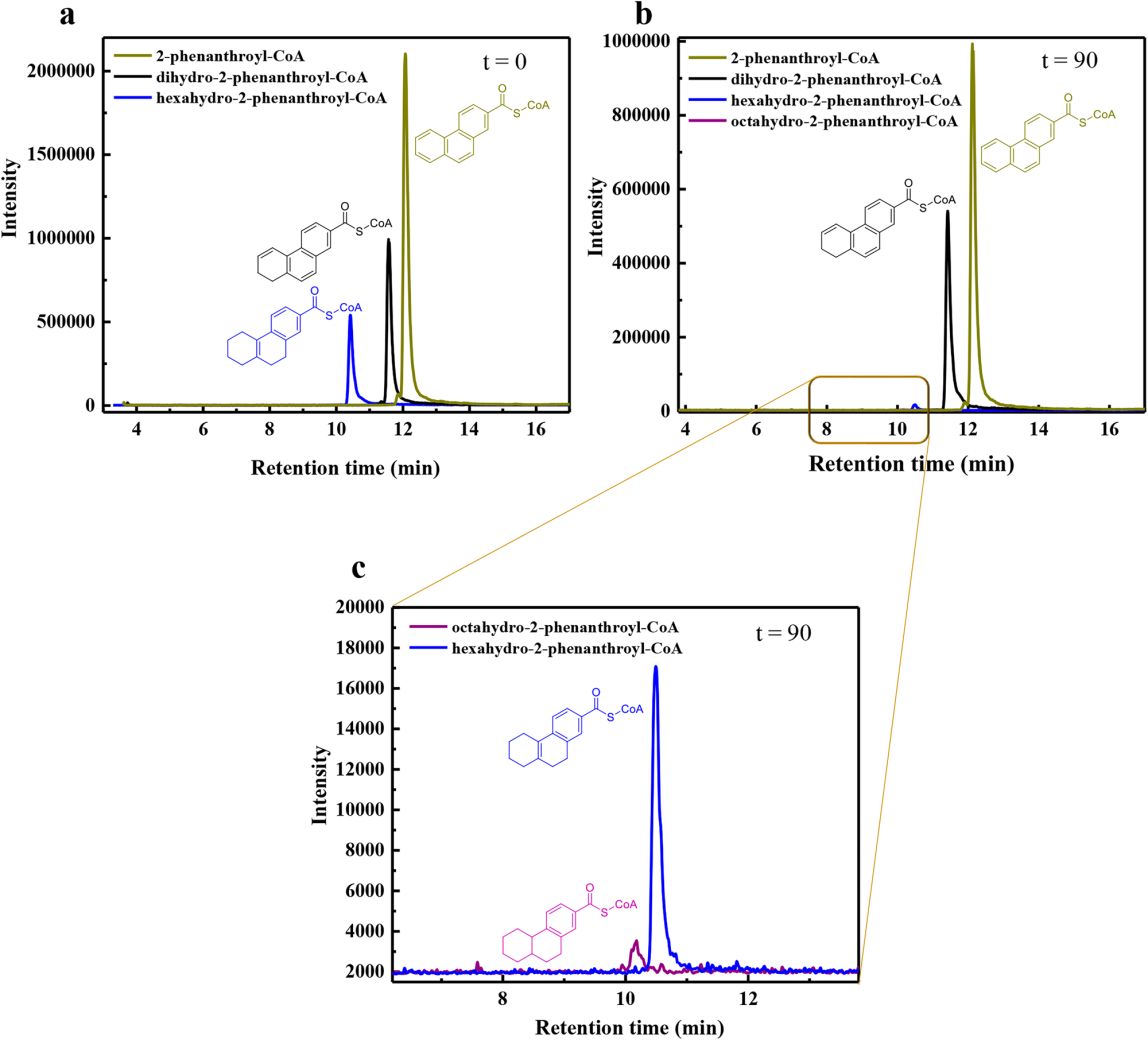
LC-MS analysis showing the in vitro conversion of the enzymatically accumulated hexahydro-2-phenanthroyl-CoA (6) (substrate) to the reduced product, octahydro-2-phenanthroyl-CoA (7). Graph (**a**) at 0 min shows the enzymatic conversion of chemically synthesized 2-phenanthroyl-CoA (**3**) to hexahydro-2-phenanthroyl-CoA (**6**) by 2-phenanthroyl-CoA reductase (AprB) and dihydro-2-phenanthroyl-CoA reductase (AprC). Graph (**b**) at 90 min shows the results of the two-electron reduction reaction of hexahydro-2-phenanthroyl-CoA (**6**) (substrate) catalyzed by hexahydro-2-phenanthroyl-CoA reductase (AprD), producing octahydro-2-phenanthroyl-CoA (**7**) (purple peak in the enlarged graph (**c**), m/z = 980, positive ion mode). The yellow line in graph (**a**) and (**b**) indicates the leftover of the chemically synthesized 2-phenanthroyl-CoA (**3**) (m/z = 972, positive ion mode). The black line in graph (**a**) and (**b**) indicates the leftover of the enzymatically accumulated dihydro-2-phenanthroyl-CoA (**4a or 4b**) (m/z = 974, positive ion mode). The blue line in the three graphs depicts the substrate, hexahydro-2-phenanthroyl-CoA (**6**) (m/z = 978, positive ion mode). In graph (**c**), the peaks of 2-phenanthroyl-CoA (**3**) and dihydro-2-phenanthroyl-CoA (**4a or 4b**) were hidden due to their high intensity, which prevented the visualization of the much lower peaks of hexahydro-2-phenanthroyl-CoA (**6**) and octahydro-2-phenanthroyl-CoA (**7**).

**Fig. 5:**
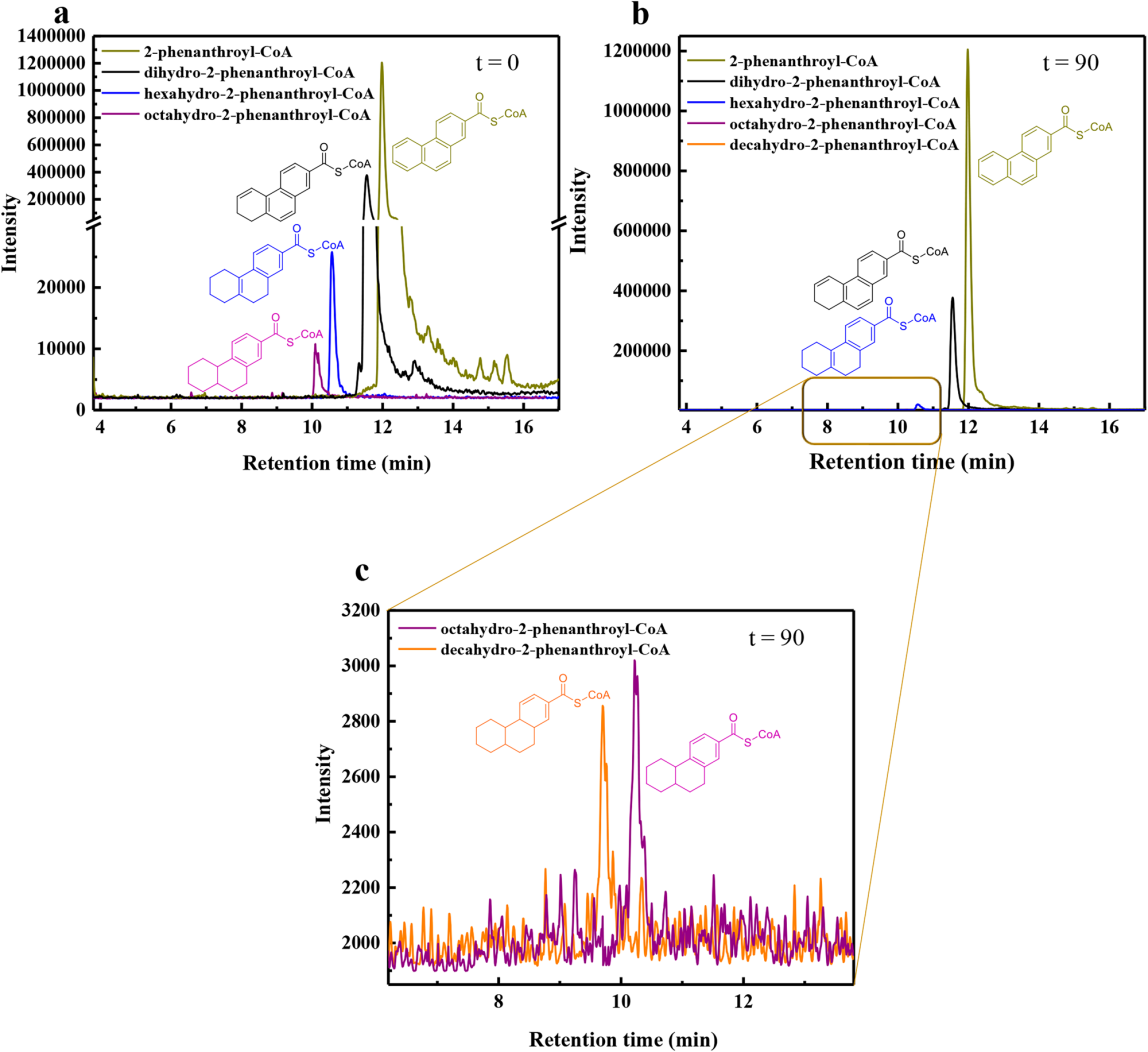
LC-MS analysis showing the in vitro conversion of enzymatically accumulated octahydro-2-phenanthroyl-CoA (7) (substrate) to the reduced product, decahydro-2-phenanthroyl-CoA (8). Graph (**a**) at 0 min shows the enzymatic accumulation of the substrate by reducing the chemically synthesized 2-phenanthroyl-CoA (**3**) using 2-phenanthroyl-CoA reductase (AprB), dihydro-2-phenanthroyl-CoA reductase (AprC), and hexahydro-2-phenanthroyl-CoA reductase (AprD). Graph (**b**) at 90 min shows the results of the two-electron reduction reaction of octahydro-2-phenanthroyl-CoA (**7**) (substrate) catalyzed by octahydro-2-phenanthroyl-CoA reductase (AprE), producing decahydro-2-phenanthroyl-CoA (**8**) (orange peak in the enlarged graph (**c**), m/z = 982, positive ion mode). The yellow line in graphs (**a**) and (**b**) shows the leftover of chemically synthesized 2-phenanthroyl-CoA (**3**) (m/z = 972, positive ion mode). The black line in graph (**a**) and (**b**) shows the leftover of the enzymatically accumulated dihydro-2-phenanthroyl-CoA (**4a or 4b**) (m/z = 974, positive ion mode). The blue line in graph (**a**) and (**b**) shows the leftover of hexahydro-2-phenanthroyl-CoA (**6**) (m/z = 978, positive ion mode). The purple line in graph (**a**) and (**c**) shows the substrate, octahydro-2-phenanthroyl-CoA (**7**) (m/z = 980, positive ion mode). In graph (**c**), the peaks of 2-phenanthroyl-CoA (**3**), dihydro-2-phenanthroyl-CoA (**4a or 4b**), and hexahydro-2-phenanthroyl-CoA (**6**) were omitted due to their high intensity which prevented the visualization of the lower peaks of octahydro-2-phenanthroyl-CoA (**7**) and decahydro-2-phenanthroyl-CoA (**8**).

The activity of hexahydro-2-phenanthroyl-CoA reductase (AprD) toward hexahydro-2-phenanthroyl-CoA (**6**) (substrate) was investigated using the purified enzyme in the presence of dithionite-reduced methyl viologen as electron donor, 50 µM FMN, and 100 µM FAD as cofactors. Due to the commercial unavailability of hexahydro-2-phenanthroyl-CoA, the compound was enzymatically accumulated by reducing the chemically synthesized 2-phenanthroyl-CoA (**3**) using 2-phenanthroyl-CoA reductase (AprB) and dihydro-2-phenanthroyl-CoA reductase (AprC), as described previously^18,19^. The hexahydro-2-phenanthroyl-CoA reductase (AprD) catalyzed a two-electron reduction step producing octahydro-2-phenanthroyl-CoA (**7**) with a specific activity of 12.7 nmol min^-1^ mg^-1^ (Fig. 4), and an apparent *K*_M_ value of 15.1 nM (Table 1). It might be possible that the residual substrates might have had competitive inhibitory effects on the enzyme kinetics. Octahydro-2-phenanthroyl-CoA (**7**) was accumulated enzymatically in large amounts to be used as a substrate for octahydro-2-phenanthroyl-CoA reductase (AprE). The stock concentration of octahydro-2-phenanthroyl-CoA (**7**) was quantified and used to prepare initial concentrations ranging from 1 to 250 nM to determine the initial rate of octahydro-2-phenanthroyl-CoA (**7**) reduction to decahydro-2-phenanthroyl-CoA (**8**). Octahydro-2-phenanthroyl-CoA reductase (AprE) catalyzed a two-electron reduction step producing decahydro-2-phenanthroyl-CoA (**8**) with a specific activity of 6.3 nmol min^-1^ mg^-1^ (Fig. 5) and an apparent *K*_M_ value of 63.9 nM (Table 1). Notably, the two reduction steps of hexahydro-2-phenanthroyl-CoA (**6**) to decahydro-2-phenanthroyl-CoA (**8**) catalyzed by both AprD and AprE were ATP-independent. Addition of ATP did not affect the reaction rate, confirming that hexahydro-2-phenanthroyl-CoA reductase (AprD) and octahydro-2-phenanthroyl-CoA reductase (AprE) belong to the ATP-independent class III of aryl-CoA reductases. The enzymatic reduction assays with AprD and AprE had to be conducted in an anaerobic chamber to prevent chemical oxidation of the electron donor, dithionite-reduced methyl viologen. Nonetheless, neither enzyme is sensitive to oxygen, as they were active although overproduced and purified under atmospheric conditions. This resembles 2-naphthoyl-CoA reductase and dihydro-2-naphthoyl-CoA reductase, which reduce 2-naphthoyl-CoA to 5,6-dihydro-2-naphthoyl-CoA and 5,6,7,8-tetrahydro-2-naphthoyl-CoA, respectively, in the sulfate-reducing Deltaproteobacteria N47 and strain NaphS2^15,17^. However, not all OYEs are insensitive to oxygen^31^. Different alternative electron donors (dithionite, NADH, Ti(III)-citrate, and NADPH) were tested to check their effect on the catalytic activities of hexahydro-2-phenanthroyl-CoA reductase (AprD) and octahydro-2-phenanthroyl-CoA reductase (AprE). Only hexahydro-2-phenanthroyl-CoA reductase (AprD) showed 5% of activity when NADH was used as the sole electron donor compared to dithionite-reduced methyl viologen. The function of NADH in the enzymatic assays with dithionite-reduced methyl viologen may be to keep the flavins in a reduced state^32^. The enzyme did not show activity with only dithionite, Ti(III)-citrate, or NADPH, as electron donors. Octahydro-2-phenanthroyl-CoA reductase (AprE) was only active with dithionite-reduced methyl viologen as an electron donor and did not show catalytic activity with other electron donors. Q-TOF/LC-MS confirmed the structures of the reduced products, octahydro-2-phenanthroyl-CoA (**7**) and decahydro-2-phenanthroyl-CoA (**8**) as C_15_H_17_O-CoA and C_15_H_19_O-CoA, respectively.

### Enoyl-CoA hydratase (ApcA) adds a water molecule to one of the two double bonds of the cyclic diene decahydro-2-phenanthroyl-CoA

**Fig. 6:**
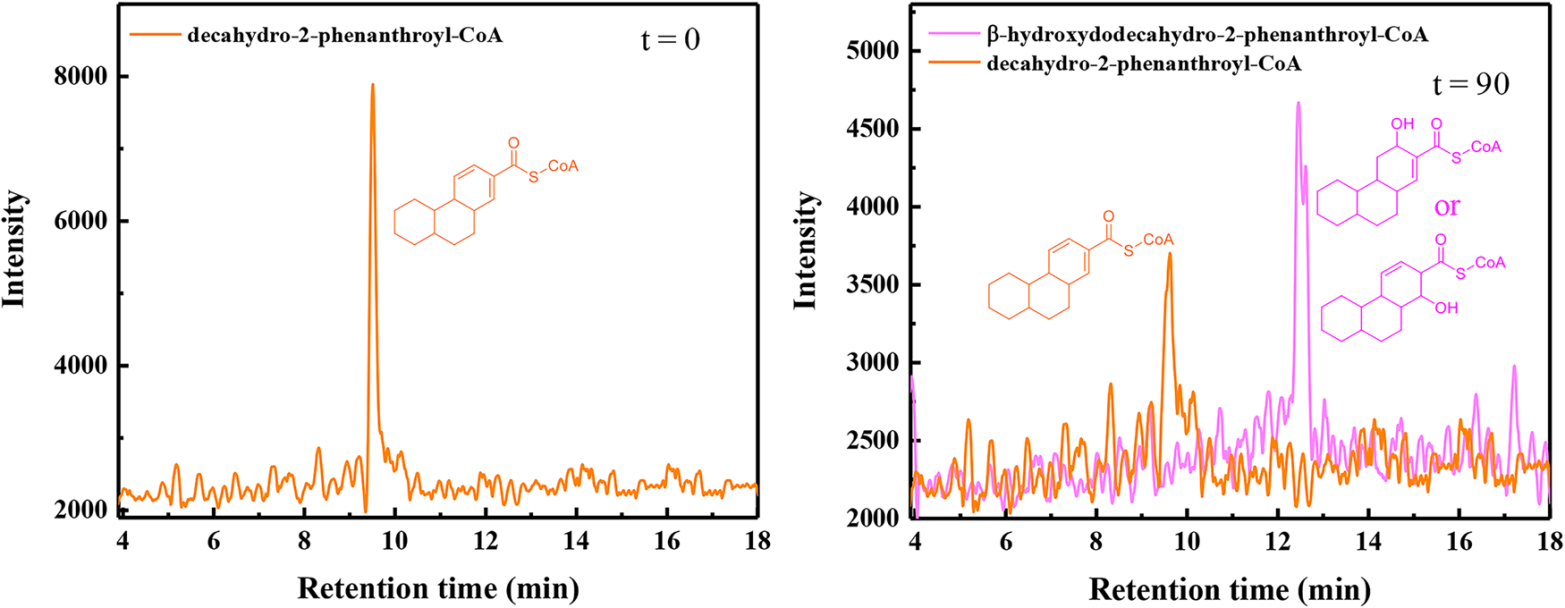
LC-MS analysis showing the in vitro conversion of enzymatically accumulated decahydro-2-phenanthroyl-CoA (8) (substrate) to β-hydroxydodecahydro-2-phenanthroyl-CoA (9) by enoyl-CoA hydratase (ApcA). The graph at 0 min shows enzymatic accumulation of the substrate by reducing octahydro-2-phenanthroyl-CoA (**7**) to decahydro-2-phenanthroyl-CoA (**8**) (orange line, m/z = 982, positive ion mode) using AprE. The graph at 90 min shows the results of adding one water molecule catalyzed by enoyl-CoA hydratase (ApcA), producing one of two possible isomers of β-hydroxydodecahydro-2-phenanthroyl-CoA (**9**) (magenta line, m/z = 1000, positive ion mode). The position of the double bonds in the structures of decahydro-2-phenanthroyl-CoA (**8**) and β-hydroxydodecahydro-2-phenanthroyl-CoA (**9**) are shown exemplarily since the exact isomeric structures are not known.

Decahydro-2-phenanthroyl-CoA (**8**) was enzymatically accumulated after reducing the octahydro-2-phenanthroyl-CoA (**7**) by octahydro-2-phenanthroyl-CoA reductase (AprE) and used as substrate. ApcA catalyzed the conversion of decahydro-2-phenanthroyl-CoA (**8**) to one of two possible isomers of the β-hydroxydodecahydro-2-phenanthroyl-CoA (**9**) with a specific activity of 3.2 nmol min^-1^ mg^-1^ (Fig. 6) and an apparent *K*_M_ value of 31.5 nM (Table 1) at pH value of 7.5. The catalytic activity of the enoyl-CoA hydratase ApcA was oxygen insensitive and the reaction did not need cofactors or metal ions. Q-TOF/LC-MS confirmed the structure of β-hydroxydodecahydro-2-phenanthroyl-CoA (**9**) as C_15_H_21_O_2_-CoA.

### Profiling of 2-phenanthroyl-CoA degradation metabolites using culture TRIP 1 cell-free extract

**Fig. 7:**
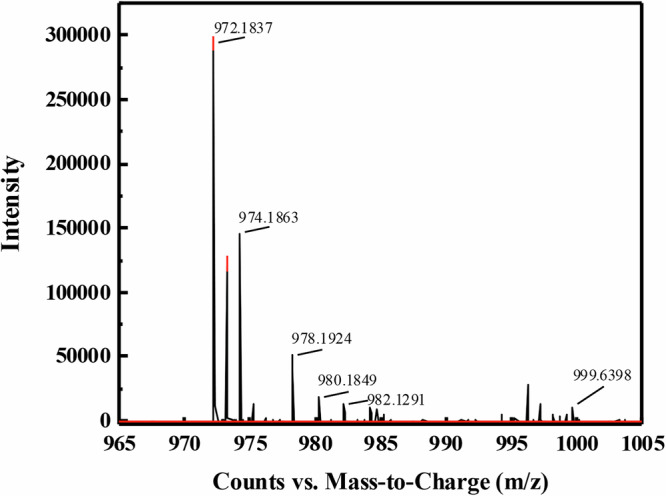
Q-TOF/LC-MS analysis (positive ion mode) of the degradation metabolites produced after reacting 2-phenanthroyl-CoA (3) with culture TRIP 1 cell-free extract and enoyl-CoA hydratase (ApcA) for 90 min. The spectra show the [M + H]^+^ signals of 2-phenanthroyl-CoA (**3**) (substrate, m/z = 972.1837), dihydro-2-phenanthroyl-CoA (**4a or 4b**) (m/z = 974.1863), hexahydro-2-phenanthroyl-CoA (**6**) (m/z = 978.1924), octahydro-2-phenanthroyl-CoA (**7**) (m/z = 980.1849), decahydro-2-phenanthroyl-CoA (**8**) (m/z = 982.1291), and β-hydroxydodecahydro-2-phenanthroyl-CoA (**9**) (m/z = 999.6398). The signal of β-hydroxydodecahydro-2-phenanthroyl-CoA (**9**) at m/z = 999.6398 measured with the high-resolution Q-TOF/LC-MS corresponds to the signal of the same compound shown at m/z = 1000 when using low-resolution LC-MS. The mass difference of 0.4 Da is within the acceptable tolerance range (≤ 0.5 Da) and is attributed to the lower mass resolution of the single quadrupole instrument, which reports nominal integer masses, compared to the high-resolution Q-TOF.

2-Phenanthroyl-CoA (**3**) (m/z = 972.1837) was reacted with culture TRIP 1 cell-free extract and enoyl-CoA hydratase (ApcA) to detect the reduction metabolites and the formation of the first product of the β-oxidation degradation pathway, i.e., β-hydroxydodecahydro-2-phenanthroyl-CoA (**9**). All the corresponding mass-to-charge ratio of dihydro-2-phenanthroyl-CoA (**4a or 4b**) (m/z = 974.1863), hexahydro-2-phenanthroyl-CoA (**6**) (m/z = 978.1924), octahydro-2-phenanthroyl-CoA (**7**) (m/z = 980.1849), decahydro-2-phenanthroyl-CoA (**8**) (m/z = 982.1291), and β-hydroxydodecahydro-2-phenanthroyl-CoA (**9**) (m/z = 999.6398) were detected (Fig. 7). The signal of β-hydroxydodecahydro-2-phenanthroyl-CoA (**9**) at m/z = 999.6398 measured with the high-resolution Q-TOF/LC-MS corresponds to the signal of the same compound shown at m/z = 1000 when using low-resolution LC-MS. These results confirm that the enzymatic reduction pathway studied with heterologously expressed type III aryl-CoA reductases takes place in the cells^1,18,19,25^. Moreover, mass spectrometric analysis indicated that the β-oxidation-like pathway starts with a hydratase reaction by adding a water molecule to the diene, decahydro-2-phenanthroyl-CoA (**8**) to produce the β-hydroxydodecahydro-2-phenanthroyl-CoA (**9**).

## Discussion

The anaerobic degradation of aromatic hydrocarbons comprises three essential biochemical problems: the activation of the chemically inert compounds, which can be performed, for example, by fumarate addition to methyl groups, or a carboxylation of the aromatic ring in the case of benzene and non-substituted PAHs^1,4,23,33^. The second challenge is the dearomatization of the ring system, which is achieved via two-electron reduction. The third challenge is the ring cleavage, which can either be performed by hydrolysis, as in the case of the benzoyl-CoA pathway of monoaromatic compounds, or by so far unknown lyase reactions in the case of naphthalene^1,34^.

As an example of anaerobic phenanthrene degradation, we elucidated here how bigger PAHs with three or more rings get dearomatized by reduction reactions. The only comparable example investigated so far is the anaerobic degradation of the two-ring PAH naphthalene. Here, the reduction of naphthoyl-CoA starts with two ATP-independent two-electron reduction steps catalyzed by type III aryl-CoA reductases of the OYE family and producing tetrahydro-2-naphthoyl-CoA, which still contains one remaining benzene ring in proximity to the carboxyl group. This benzene ring is then reduced by an ATP-dependent type I aryl-CoA reductase, which is similar to benzoyl-CoA reductase of the *Thauera* type. Here, we could show that in anaerobic phenanthrene degradation, there is no such ATP-dependent reduction step involved, but the full dearomatization of the phenanthroyl-CoA ring system is performed by four ATP-independent type III aryl-CoA reductases producing the diene compound decahydro-2-phenanthoyl-CoA. We also demonstrated that this diene is the direct substrate for the subsequent hydratase reaction, proving that the reduction pathway is complete and lacks no other, as yet uninvestigated, enzymes.

In this study, we provide biochemical in vitro evidence for the complete reduction of the aromatic rings of 2-phenanthroyl-CoA (**3**) during anaerobic degradation of phenanthrene. Previously, we showed that 2-phenanthroyl-CoA (**3**) is reduced to dihydro-2-phenanthroyl-CoA [4a or 4b] by the ATP-independent 2-phenanthroyl-CoA reductase (AprB)^18^. The next reduction step was catalyzed by the ATP-independent dihydro-2-phenanthroyl-CoA reductase (AprC), which performs two consecutive two-electron reduction steps (a four-electron reduction in total), converting the 5,6-dihydro-2-phenanthroyl-CoA(**4b**) or 7,8-dihydro-2-phenanthroyl-CoA(**4a**) to hexahydro-2-phenanthroyl-CoA (**6**)^19^. The produced hexahydro-2-phenanthroyl-CoA (**6**) can theoretically occur in eight possible isomers; three of these isomers (isomer 6f–h, Supplementary Fig. 2) have conjugated double bonds and no benzene rings, and the other five have one benzene ring and one double bond arranged in different positions (isomer 6b–e, Supplementary Fig. 2)^19^. Both types of structures allow for a two-electron reduction step of hexahydro-2-phenanthroyl-CoA (**6**) to octahydro-2-phenanthroyl-CoA (**7**) by hexahydro-2-phenanthroyl-CoA reductase (AprD) without energy input via ATP hydrolysis.

Octahydro-2-phenanthroyl-CoA (**7**) is then reduced by octahydro-2-phenanthroyl-CoA reductase (AprE) to decahydro-2-phenanthroyl-CoA (**8**), which is a diene compound with no aromatic ring left. Hence, anaerobic phenanthrene degradation entails full dearomatization of phenanthroyl-CoA to a diene, without the expense of ATP hydrolysis.

AprD and AprE were characterized, and both were found to belong to the type III aryl-CoA reductases of the OYE family with high amino acid similarities of 30–34% with 2-phenanthroyl-CoA reductase (AprB) and dihydro-2-phenanthroyl-CoA reductase (AprC)^18,19^. Moreover, *aprD* and *aprE* showed 34-37% amino acid similarity with the amino acid sequence of 2-naphthoyl-CoA reductase encoded by genes N47_G38220, N47_G38210, NPH_5475, NPH_1753, and NPH_5476 in the naphthalene-degrading strains N47 and NaphS2^27,29,35^.

The last reduction step of octahydro-2-phenanthroyl-CoA (**7**) to decahydro-2-phenanthroyl-CoA (**8**) is of particular interest because it is similar to the two-electron reduction of tetrahydro-2-naphthoyl-CoA to hexahydro-2-naphthoyl-CoA, or benzoyl-CoA to cyclohexa-1,5-diene-1-carboxyl-CoA, which are catalyzed by ATP-dependent and oxygen-sensitive type I aryl-CoA reductase of the benzoyl-CoA reductase type from the facultative anaerobic bacteria *Thauera* or *Azoarcus*^36^. So, the question arises how this difficult reduction can be performed in anaerobic phenanthrene degradation without ATP-hydrolysis.

**Fig. 8:**
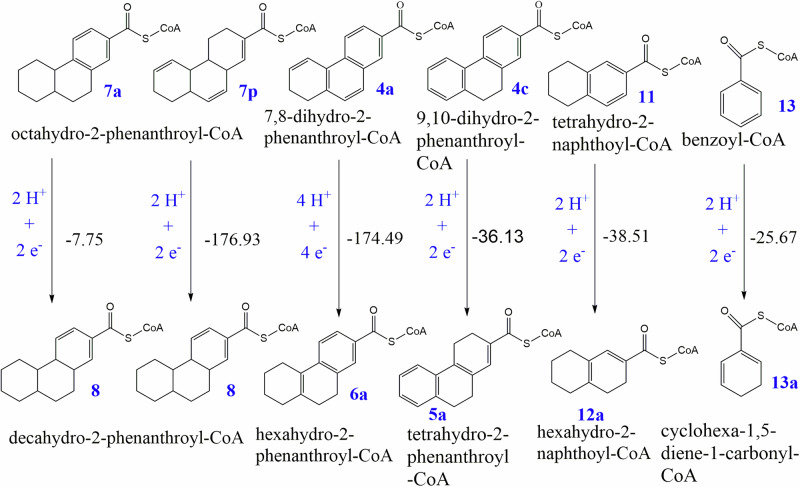
Gibbs free energy changes (ΔG_298_) between the substrates (**7a**), (**7p**), (**4a**), (**4c**), (**11**), and (**13**) and their corresponding products (**8**), (**6a**), (**5a**), (**12a**), and (**13a**), respectively. ΔG298 (at standard conditions but 298 K and pressure 1 bar [kJ mol-1]) was calculated at the B3LYP/6-311+G(d,p) level of theory. The image suggests that reduction of (**7p**) to (**8**) and reduction of (**4a**) to (**6a**) using type III aryl-CoA reductases are easier compared to other reductions in the image because of the more negative ΔG. Detailed ΔG_298_ calculation for other octahydro-2-phenanthroyl-CoA (**7**) isomers can be found in Table S1.

The benzoyl-CoA reduction (before ATP coupling ΔG −25.67 kJ mol^-1^, Fig. 8) couples the transfer of two electrons to a stoichiometric hydrolysis of two molecules ATP to ADP + Pi and requires a low-potential ferredoxin as electron donor^37,38^. The coupling of ring reduction to the hydrolysis of two ATP molecules renders the overall reaction essentially irreversible (ΔG^0’^ ≈ −60 kJ mol^-1^ at physiological conditions in the cell)^39^. This energy input is obviously high enough to overcome the activation energy of the reduction of the benzene ring, but there is no such energy input in the reduction of octahydro-2-phenanthroyl-CoA to decahydro-2-phenanthroyl-CoA (Fig. 8). Both types of reduction reactions need a similarly low potential electron donor in the form of methyl viologen in the in vitro tests indicating that the reduction reactions most likely occur with a ferredoxin as electron donor in vivo. Furthermore, the total free ∆G of the reduction reactions is more or less the same for the two types of reactions^19^. We conclude that the difference is in the different activation energies for the reduction of the substrates (Fig. 8).

**Fig. 9:**
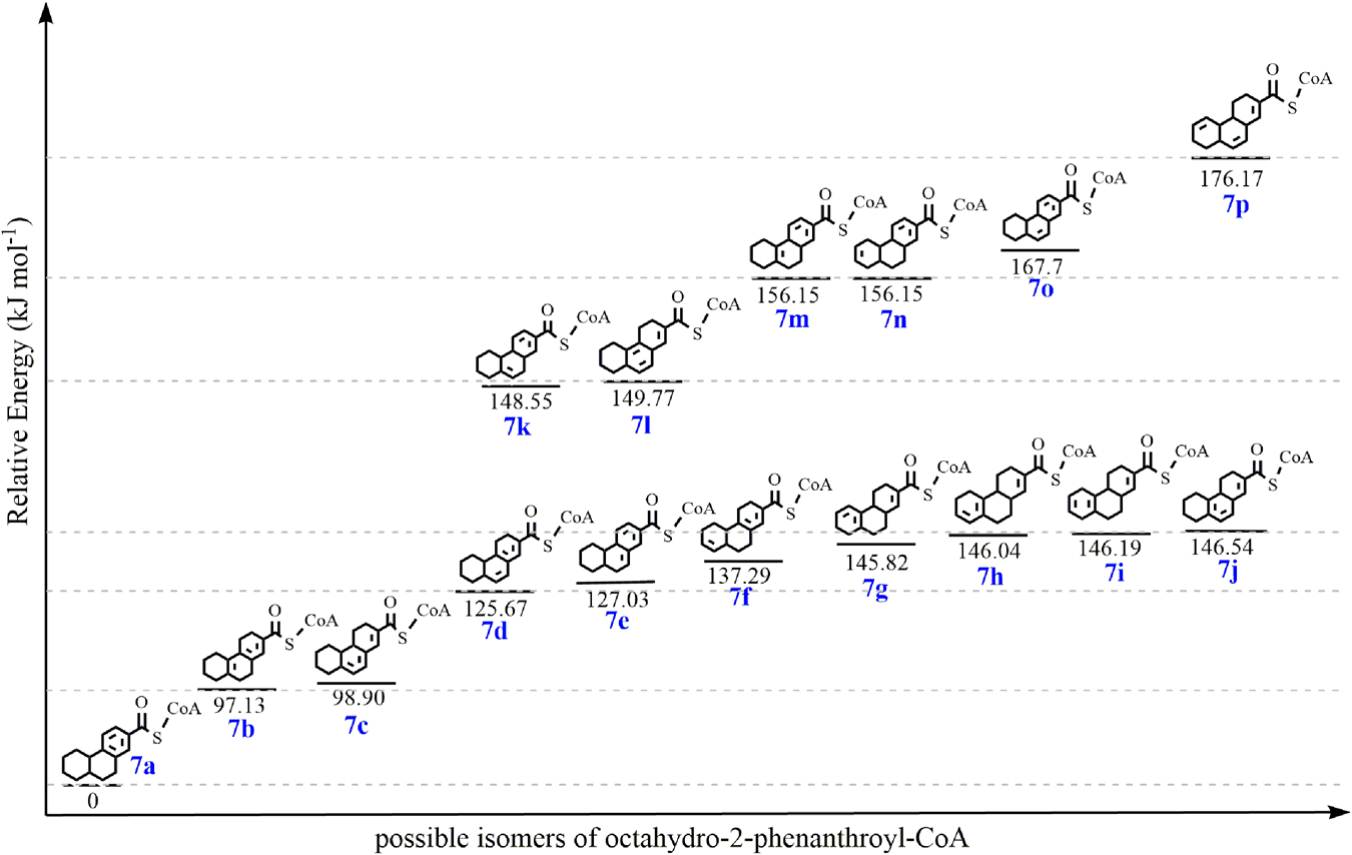
Relative electronic energies [kJ mol^-1^] of sixteen possible octahydro-2-phenanthroyl-CoA (**7**) isomers. The relative electronic energies were calculated at the B3LYP/6-311 + G(d,p) level of theory.

So far, we do not know the exact electronic structures of the metabolites in the 2-phenanthroyl-CoA reduction pathway; nevertheless, we propose two different possible scenarios for the reduction: *i*. octahydro-2-phenanthroyl-CoA has a benzene ring, which has to be reduced (compound **7a** in Fig. 9), or *ii*. octahydro-2-phenanthroyl-CoA has a non-aromatic but still conjugated double-bond system (*e. g*., compounds **7b–p** in Fig. 9). *i*. This option, where the substrate contains a benzene ring, is supported by previous results^19^ where the benzene ring III of 9,10-dihydro-2-phenanthroyl-CoA (**4c**) could be reduced by the type III dihydro-2-phenanthroyl-CoA reductase (AprC), although this ring was not fully conjugated to the other benzene ring I because they are separated by a sigma bond. However, the 3D structure of compound (**4c**) is still close to coplanar, still allowing for a certain interaction of the π-electron systems of the two benzene rings. The three-ring structure also forces the sp^3^ hybridized carbon centers to be asymmetric, which in total might lower the activation energy needed for reduction by the ATP-independent type III aryl-CoA reductase AprB^19^. Hence, it could be that the ring structure of octahydro-2-phenanthroyl-CoA (**7**) contains a benzene ring, but the structural distortion of the carbon centers lowers the activation energy of the reaction such that it can be reduced by the type III aryl-CoA reductase AprE without the expense of ATP-hydrolysis. A major argument against this hypothesis is the energy calculation of the different possible isomers of octahydro-2-phenanthroyl-CoA (**7**) (Fig. 9 and Table S1). The only possible isomer containing a benzene ring(**7a**) has a 97.13–98.90 kJ mol^-1^ lower electronic energy compared to isomers (**7b-e**), which have a conjugated but not aromatic double bond system. *ii*, If octahydro-2-phenanthroyl-CoA reductase AprD produces a non-aromatic isomer with conjugated double bonds instead of one containing a benzene ring, the last two-electron reduction step would be much easier and could explain why this reduction step does not require additional energy input by ATP-hydrolysis. This fact is also supported by the ΔG calculation (Table S1) for isomers (**7b-e**), which is ≈ -96.17-176.93 kJ mol^-1^ compared to isomer (**7a**) (ΔG −7.75 kJ mol^-1^). Isomers of octahydro-2-phenanthroyl-CoA, such as (**7b-e**) may quickly rearomatize to isomer (**7a**) if stored in aqueous or organic solvents, due to the higher stability of the benzene ring. However, in our enzyme assays and especially in the living cell, the half-life time of a non-aromatic substrate might be long enough to be reduced by the last reductase, octahydro-2-phenanthroyl-CoA reductase AprE, producing the stable diene, which can be further hydrated.

After the last two-electron reduction step of octahydro-2-phenanthroyl-CoA (**7**) to the diene decahydro-2-phenanthroyl-CoA (**8**) catalyzed by AprE, we added the heterologously expressed and purified enoyl-CoA hydratase ApcA, which hydrated the substrate (**8**) to β-hydroxydodecahydro-2-phenanthroyl-CoA (**9**). This step is very important because it demonstrates that the reduction of the phenanthroyl-CoA ring skeleton is completed by the production of the diene and requires no additional enzymes beyond the four type III aryl-CoA reductases, AprB-E, and a suitable electron donor, most likely ferredoxin in vivo. Furthermore, the hydratase ApcA is the first enzyme in the β-oxidation pathway that ultimately leads to ring cleavage and release of acetyl-CoA units to enter the central metabolism.

The enoyl-CoA hydratase ApcA belongs to the crotonase superfamily, which is known to catalyze the addition of water to a trans-2-enoyl-CoA thioester, producing a β-hydroxyacyl-CoA thioester without the assistance of any cofactors or metal ions in the β-oxidation pathway of fatty acid metabolism^40,41^. Enzymes of the crotonase family are involved in the degradation or the biosynthesis of various enoyl compounds and fatty acids in all organisms^42^.

The amino acid sequence of enoyl-CoA hydratase (*apcA*) showed high similarity with the amino acid sequence of enoyl-CoA hydratase/hydrolase/isomerase encoded by the genes N47_E41500 (60%), N47_E41380 (41%), N47_E41370 (40%), NPH_5886 (57%), and NPH_5907 (42%) from the *Deltaproteobacteria* N47 and NaphS2^27,29,35^. Enoyl-CoA hydratase acts by a catalytic acid and base mechanism due to the presence of two glutamate residues in the enzyme and facilitates the addition of a water molecule to the double bond of enoyl-CoA substrates^42,43^. The mechanism of action of the homodimeric enoyl-CoA hydratase (ApcA) is believed to be similar to the homodimeric dienoyl-CoA hydratase in the denitrifying bacterium *Thauera aromatica,* which has a subunit molecular mass of 28 kDa and native molecular mass of 55 kDa^44^. Dienoyl-CoA hydratase in the denitrifying bacterium *Thauera aromatica* catalyzes the addition of water to cyclohexa-1,5-diene-1-carboxyl-CoA, producing 6-hydroxycyclohex-1-ene-1-carboxyl-CoA^44^. It also catalyzes the direct hydrolysis of 6-hydroxycyclohex-1-ene-1-carboxyl-CoA in a coupled hydratase/hydrolase reaction, but such a hydrolase activity was not observed for ApcA.

## Conclusion

The reduction of the fully aromatic metabolite 2-phenanthroyl-CoA proceeds solely through four ATP-independent type III aryl-CoA reductases (AprB-E) and produces the non-aromatic diene decahydro-2-phenanthroyl-CoA (**8**). This is furthermore supported by the conversion of the diene metabolite (**8**) by the next enzyme in the pathway, the enoyl-CoA hydratase ApcA, which adds a water molecule, producing β-hydroxydodecahydro-2-phenanthroyl-CoA (**9**), indicating that no other enzymes are needed for the complete reduction to the diene.

Hence, the reduction pathway in anaerobic phenanthrene degradation comprising only four ATP-independent type III aryl-CoA reductases constitutes a new strategy of overcoming the resonance energy of aromatic hydrocarbons in anaerobic degradation besides the two types of benzoyl-CoA reductases for monoaromatic compound degradation (*Thauera* type I benzoyl-CoA reductase, and *Geobacter* type II benzoyl-CoA reductase), and the 2-naphthoyl-CoA reduction pathway comprising both two ATP-independent type III aryl-CoA reductases and one ATP-dependent type I aryl-CoA reductase (*Thauera* type).

## Methods

### Cloning and recombinant overproduction of AprD, AprE, and ApcA

The *aprD* (PITCH_a420108) and *aprE* (PITCH_a190075) gene sequences encoding hexahydro-2-phenanthroyl-CoA reductase and octahydro-2-phenanthroyl-CoA reductase, respectively, and the *apcA* (PITCH_a1910016) gene sequence encoding the enoyl-CoA hydratase from culture TRIP 1 were heterologously expressed in *Escherichia coli*. The sequences of the three genes are provided in the supplementary materials (Supplementary Fig. 3, S4, & S5). The *aprD* and *aprE* genes were amplified from pEX-K248 plasmids carrying the codon-optimized *aprD* and *aprE* genes, separately, and the *apcA* gene was amplified from genomic DNA of culture TRIP 1 by PCR using a 2x KAPA HiFi HotStart ready mix (Fisher Scientific GmbH, Schwerte, Germany), with the corresponding forward and reverse primers of each gene (Table S2). The PCR products were then purified separately using the Monarch DNA Gel Extraction Kit (New England Biolabs GmbH, Massachusetts, USA). The pure PCR products were integrated with the expression plasmid, pASG-IBA103, as following: the purified PCR products (2 nM) were digested with the restriction enzyme Esp3I (2.5 µL, 5 U), and the genes were each ligated into a pASG-IBA103 expression plasmid (5 ng) using 250 mM DTT/12.5 mM ATP mix (1 µL) and T4 DNA ligase (1 U), followed by incubation for 1 h at 30 °C. The resulting recombinant constructs, pASG-IBA103-*aprD*, pASG-IBA103-*aprE*, and pASG-IBA103- *apcA*, were first transformed into NEB-5α competent *E. coli* cells, then isolated and transformed into BL21(DE3) *E. coli* cells for heterologous overproduction. The recombinant plasmids were extracted and sequenced to confirm successful cloning (Eurofins Genomics, Ebersberg, Germany).

To overproduce the hexahydro-2-phenanthroyl-CoA reductase (AprD), octahydro-2-phenanthroyl-CoA reductase (AprE), and the enoyl-CoA hydratase (ApcA) enzymes, *E. coli* BL21(DE3) was transformed with the recombinant plasmids pASG-IBA103-*aprD*, pASG-IBA103-*aprE*, and pASG-IBA103-*apcA* separately, and cells were grown in a 1 L flask with 500 mL Lysogeny Broth (LB) supplemented with ampicillin (100 µg/mL) and approximately 0.3 g riboflavin in case of AprD and AprE, and supplemented only with ampicillin (100 µg/mL) in case of ApcA. The cultures were incubated at 37 °C in Erlenmeyer flasks and shaken at 200 rpm until the optical density of the cultures reached an optical density of 0.5–0.6 at 600 nm. The three enzymes were induced with anhydrotetracycline (0.2 µg/mL) followed by incubation at 20 °C for 20 h at 130 rpm. Cells were harvested by centrifugation (3100 x g, 30 min), and the pellets were resuspended in 50 mM HEPES buffer (pH 8, 2 mL buffer per gram wet cell weight). All cultures produced ≈ 10–12 g wet cell weight per litre. Cells were disrupted using a French press at 83 bar and cell debris was removed by centrifugation (16,000 x g, one hour) in 2 mL tubes at 4 °C. The supernatants (≈ 20-30 mL) of AprD, AprE, and ApcA were applied to gravity flow Strep-Tactin® XT 4Flow® columns (IBA-lifesciences, Göttingen, Germany) and eluted with 50 mM biotin in 50 mM HEPES buffer, containing 150 mM NaCl, collecting three fractions of 3, 8, and 4 mL for each enzyme, according to IBA-lifesciences instructions. The purified proteins of AprD and AprE were concentrated using a Pierce™ Protein Concentrator (Thermo Fisher Scientific, Massachusetts, USA) with a 50 kDa cut-off, while the purified protein of ApcA was concentrated with a 10 kDa cut-off membrane.

Protein concentrations were measured with the Bradford assay^45^, and the purity of each enzyme was evaluated by SDS-PAGE using 12% Mini-PROTEAN® TGX gels (BioRad, USA). The molecular masses of AprD and AprE were determined by comparison with the Thermo Scientific PageRuler Prestained Protein Ladder, and the molecular mass of ApcA was determined with Low-Range SDS-PAGE Standard (Biorad, Germany).

### Determination of flavin and iron content in AprD and AprE reductases

The presence of flavins in hexahydro-2-phenanthroyl-CoA reductase (AprD) and octahydro-2-phenanthroyl-CoA reductase (AprE) was detected using UV/vis absorption spectroscopy on an Infinite M200 Pro TECAN Spectrophotometer (Tecan Group Ltd., Switzerland). The purified enzymes (20 µM) were placed separately in nitrogen-flushed, stoppered quartz cuvettes and reduced by adding 0.15 mM sodium dithionite. For comparison, the UV/vis spectra were also recorded for enoyl-CoA hydratase (ApcA) without the addition of a reducing agent. The flavin content of both AprD and AprE was then determined by flavin extraction and LC-MS quantification from three or four independent protein samples, as previously described^5,18^. Values are reported as mean ± Standard Deviation (SD) from three or four independent biological replicates, calculated from LC-MS quantification of extracted flavins normalized to enzyme concentration (Table S3). Iron content was measured according to refs. ^18,19^ using a ferrozine-based colorimetric assay adapted from ref. ^46^.

### Enzymatic synthesis of CoA-thioester substrates

Substrates used in this study were 2-phenanthroyl-CoA (**3**), hexahydro-2-phenanthroyl-CoA, octahydro-2-phenanthroyl-CoA (**7**), and decahydro-2-phenanthroyl-CoA (**8**). 2-Phenanthroyl-CoA (**3**) was chemically synthesized and purified as described previously^18^. The other three substrates were not commercially available and were synthesized enzymatically. Dihydro-2-phenanthroyl-CoA (**4a or 4b**) was enzymatically synthesized by reducing the chemically synthesized 2-phenanthroyl-CoA (**3**) with 2-phenanthroyl-CoA reductase (AprB) as described previously^18^. The enzymatically formed dihydro-2-phenanthroyl-CoA (**4a or 4b**) served as a substrate for the four-electron reduction with dihydro-2-phenanthroyl-CoA reductase (AprC), producing hexahydro-2-phenanthroyl-CoA (**6**) as described previously^19^. After producing the hexahydro-2-phenanthroyl-CoA (**6**), the enzyme reaction was stopped by adding a double volume of methanol, followed by centrifugation to remove precipitated protein and salts. The protein-free supernatant was transferred to a clean tube, and the methanol was evaporated with a speedDry Vacuum Concentrator (Martin Christ Gefriertrocknungsanlagen GmbH, Osterode am Harz, Germany), leaving only the aqueous phase containing hexahydro-2-phenanthroyl-CoA plus the not fully converted substrates 2-phenanthroyl-CoA and dihydro-2-phenanthroyl-CoA. The purification of hexahydro-2-phenanthroyl-CoA only was not possible. This solution was lyophilized overnight (Martin Christ Gefriertrocknungsanlagen GmbH, Osterode am Harz, Germany), and the resulting product, hexahydro-2-phenanthroyl-CoA, was then used as a substrate for hexahydro-2-phenanthroyl-CoA reductase (AprD).

### Determination of hexahydro-2-phenanthroyl-CoA reductase (AprD) activity

To determine the reduction activity of the hexahydro-2-phenanthroyl-CoA reductase (AprD), a 200 µL assay was prepared with 25 µM hexahydro-2-phenanthroyl-CoA thioester (**6**), 1 mM NADH, 50 µM FMN, 100 µM FAD, 1 mM methyl viologen, 1 mM dithionite, and 5 µg purified AprD in 50 mM HEPES buffer, pH 7.5. The reaction was incubated at 30 °C and 900 rpm in a Thermomix Block (ThermoMixer® C, Eppendorf, Germany) within a nitrogen-filled anaerobic chamber (O_2_ < 0.5 ppm) (MBraun Inertgas-Systeme GmbH, Garching, Germany). Sample aliquots (40 µL) were collected at 0, 45, and 90 min into Eppendorf tubes, mixed with double volume of methanol to stop the reaction, and centrifuged at 16,000 x g for 60 min to remove precipitates. Supernatants were analyzed by Liquid Chromatography-Mass Spectrometry (LC-MS) to measure octahydro-2-phenanthroyl-CoA thioester (**7**) production.

### Determination of octahydro-2-phenanthroyl-CoA reductase (AprE) activity

After LC-MS confirmed the successful production of octahydro-2-phenanthroyl-CoA thioester (**7**), the compound was enzymatically accumulated, the methanol was evaporated, and the remaining liquid was freeze-dried as described above. The dried compound was then used as a substrate for octahydro-2-phenanthroyl-CoA reductase (AprE). Due to incomplete reactions, parts of the previous substrates 2-phenanthroyl-CoA, dihydro-2-phenanthroyl-CoA, and hexahydro-2-phenanthroyl-CoA always remained in the assays and purification of octahydro-2-phenanthroyl-CoA was not achieved. Nevertheless, the detection of single ion traces allowed for the clear separation of compound peaks in the LC-MS.

To test the reduction activity of octahydro-2-phenanthroyl-CoA reductase (AprE), we prepared a 200 µL reaction with 10 µM octahydro-2-phenanthroyl-CoA thioester (**7**) (substrate), 1 mM NADH, 50 µM FMN, 100 µM FAD, 1 mM methyl viologen, 1 mM dithionite, and 5 µg purified AprE in 50 mM HEPES buffer (pH 7.5). The reaction was incubated at 30 °C and shaken at 900 rpm in a nitrogen-filled anaerobic chamber. At 0, 45, and 90 min, 40 µL aliquots were collected into Eppendorf tubes, and the reaction was stopped with 80 µL methanol. The aliquots were spun at 16,000 x g for 60 min and analyzed by LC-MS to measure the production of decahydro-2-phenanthroyl-CoA thioester (**8**).

Various potential electron donors were evaluated for the reduction assays catalyzed by hexahydro-2-phenanthroyl-CoA reductase (AprD) and octahydro-2-phenanthroyl-CoA reductase (AprE) including sodium dithionite (5 mM), Ti(III)-citrate (5 mM), NADPH (5 mM), and dithionite (1 mM) reduced methyl viologen (1 mM). To examine possible ATP dependence, the reaction was performed with or without ATP (5 mM–50 µM).

### Determination of enoyl-CoA hydratase (ApcA) activity

The total enzyme reaction containing the enzymatically accumulated decahydro-2-phenanthroyl-CoA thioester (**8**) was freeze-dried and used as substrate for the enoyl-CoA hydratase. The separation and purification of decahydro-2-phenanthroyl-CoA thioester (**8**) from the previous substrates (2-phenanthroyl-CoA, dihydro-2-phenanthroyl-CoA, hexahydro-2-phenanthroyl-CoA, and octahydro-2-phenanthroyl-CoA) was not possible. A 200 µL reaction assay contained 10 µM decahydro-2-phenanthroyl-CoA thioester (**8**) (substrate) and 5 µg purified ApcA in 50 mM HEPES buffer (pH 7.5). The reaction was incubated at 30 °C and shaken at 900 rpm in a nitrogen-filled anaerobic chamber. At 0, 45, and 90 min, 40 µL aliquots were collected into Eppendorf tubes, and the reaction was stopped with 80 µL methanol. Samples were then centrifuged at 16,000 x g for 60 min and analyzed by LC-MS to measure the production of β-hydroxydodecahydro-2-phenanthroyl-CoA thioester (**9**). No cofactors were added for this reaction.

### Kinetic properties calculations

Specific activities of hexahydro-2-phenanthroyl-CoA reductase (AprD), octahydro-2-phenanthroyl-CoA reductase (AprE), and enoyl-CoA hydratase (ApcA) were determined by monitoring the conversion of the substrates hexahydro-2-phenanthroyl-CoA (**6**), octahydro-2-phenanthroyl-CoA (**7**), and decahydro-2-phenanthroyl-CoA (**8**) to the corresponding products octahydro-2-phenanthroyl-CoA (**7**), decahydro-2-phenanthroyl-CoA (**8**), and β-hydroxydodecahydro-2-phenanthroyl-CoA (**9**), respectively, during the first 30 min, to obtain the reaction rate per minute. To determine the initial reaction rate, apparent Michaelis constant (*K*_M_), and maximum reaction rate (*V*_max_), enzyme assays were performed using a range of starting substrate concentrations: hexahydro-2-phenanthroyl-CoA (**6**) (1–200 nM), octahydro-2-phenanthroyl-CoA (**7**) (1–250 nM), and decahydro-2-phenanthroyl-CoA (**8**) (1–200 nM). Non-linear regression analysis was used to calculate the *V*_max_ and *K*_M_ using the Michaelis-Menten model in GraphPad Prism. Specific activity was determined for three independent experiments as the *V*_max_ value normalized to protein concentration, and the mean value was represented (Supplementary Fig. 6).

### Detection of downstream degradation metabolites from culture TRIP 1 in cell-free extract

Enrichment culture TRIP 1 (2 L) was grown anaerobically for approximately 6 months as described previously^25^. The culture supernatant was separated from the paraffin oil layer using a separation funnel inside a nitrogen-filled anaerobic chamber (O_2_ < 0.5 ppm) (MBraun Inertgas-Systeme GmbH, Garching, Germany). Cells were harvested in air-tight beakers by centrifugation (3100 x g, 60 min), and the pellet was washed first with 50 mM HEPES buffer (pH 7.5) and then resuspended in 1.6 mL of the same buffer and opened with a French press at a pressure of 69 bar. Cell-free extract supernatant was separated from the cell debris by centrifugation at 16,000 x g, 4 °C for 45 min. To detect the reduction and hydratase metabolites of 2-phenanthroyl-CoA thioester (**3**), a 400 µL reaction assay was performed containing: 100 μM 2-phenanthroyl-CoA (**3**) (substrate), 1 mM NADH, 50 µM FMN, 100 µM FAD, 1 mM methyl viologen, 1 mM dithionite, 100 µL culture TRIP 1 cell-free extract, and 50 µg purified ApcA in 50 mM HEPES buffer (pH 7.5). The reaction was incubated at 30 °C and shaken at 900 rpm in a nitrogen-filled anaerobic chamber for 90 min. 40 µL aliquots were collected into Eppendorf tubes, and the reaction was stopped with 80 µL methanol. Samples were then centrifuged at 16,000 x g for 60 min and analyzed by Liquid Chromatography/Quadrupole Time-of-Flight Mass Spectrometry (Q-TOF/LC-MS, Agilent Technologies, Santa Clara, USA).

### Liquid chromatography-mass spectrometry (LC-MS) analysis

The analyses were conducted with an LC-2040C system connected to a single quadrupole mass spectrometer (LC-MS-2020) (Shimadzu, Duisburg, Germany), as described previously^18^. The metabolites were separated on a Nucleodur C18 Gravity-SB column (250 mm length, 4.6 mm inner diameter, 5 μm particle size; Macherey-Nagel GmbH, Düren, Germany) at 35 °C. Separation was achieved using a linear gradient, beginning with 90% solvent A (0.1% [wt/vol] ammonium formate) and 10% solvent B (acetonitrile), shifting to 90% solvent B over 30 min at a flow rate of 0.4 mL/min. Compounds were ionized with electrospray ionization (ESI) in both positive and negative ionization modes. The ion spray voltage was set to 4500 V for positive mode and −4500 V for negative mode, with the system operated at 350 °C. In addition to monitoring specific molecular masses, mass-to-charge ratios between 50 and 1050 m/z were scanned with a scan speed of 15,000 u/s and event time of 0.1 second. The UV/PDA sampling frequency was at a sampling rate of 6.25 Hz. Table 2 summarizes the mass-to-charge ratios after positive ionization for all metabolites discussed in this study.

**Table 2:** Mass-to-charge ratios of metabolites discussed in this study

m/z (positive ion mode)	Metabolites
972	2-phenanthroyl-CoA **(3)**
974	dihydro-2-phenanthroyl-CoA **(4a, 4b, or 4c)** (IUPAC: 1,2-dihydro-7-phenanthroyl-CoA, 3,4-dihydro-7-phenanthroyl-CoA, or 9,10-dihydro-2-phenanthroyl-CoA)
976	tetrahydro-2-phenanthroyl-CoA **(5)** (IUPAC: 1,2,3,4-tetrahydro-7-phenanthroyl-CoA)
978	hexahydro-2-phenanthroyl-CoA **(6)** (IUPAC:1,2,3,4,9,10-hexahydro-7-phenanthroyl-CoA)
980	octahydro-2-phenanthroyl-CoA **(7)** (IUPAC: 1,2,3,4,4a,9,10,10a-octahydro7-phenanthroyl-CoA)
982	decahydro-2-phenanthroyl-CoA **(8)** (IUPAC: 1,2,3,4,4a,4b,8a,9,10,10a-decahydro-7-phenanthroyl-CoA)
1000	β-hydroxydodecahydro-2-phenanthroyl-CoA **(9)** (IUPAC: 1,2,3,4,4a,4b,7,8,8a,9,10,10a-dodecahydro-8-hydroxy-7-phenanthroyl-CoA or 1,2,3,4,4a,4b,5,6,8a,9,10,10a-dodecahydro-6-hydroxy-7-phenanthroyl-CoA)

### Q-TOF/LC-MS analysis

The Q-TOF/LC-MS system included a 1290 Infinity II Multisampler (G7167B), a 1290 High-Speed Pump (G7120A), and a 1290 MCT (G7116B), coupled to a 6546 LC/Q-TOF with a DUAL-AJS ESI source from Agilent Technologies, USA. For the LC-MS analyses, water (A) and methanol (B), each containing 0.1% (v/v) formic acid, were utilized. The Q-TOF was operated in full scan data-dependent MS/MS acquisition mode, covering a mass range from 100 to 1100 Da in both positive and negative ion modes with scan rate of 4 spectra per second (4 hz). Each measurement was conducted in triplicate to ensure accuracy and reproducibility. The ion source operational parameters were set as follows: gas temperature at 320 °C, drying gas flow at 8 L/min, nebulizer pressure at 35 psi, sheath gas temperature at 350 °C, sheath gas flow at 11 L/min, and a spray voltage of ±3500 V in both positive and negative ionization modes.

### Quantum chemical calculations

The difference in electronic energy was utilized to calculate the relative stability of various possible isomers of the reduction product, octahydro-2-phenanthroyl-CoA (**7a–p**). This value indicates the difference in total electronic energies between products and reactants. Electronic energy refers to the total energy of electrons in a molecule, determined by their interactions with the nuclei and with each other. It reflects the intrinsic, quantum-mechanical energy of a molecule and is often used to compare the relative stability of chemical species^47^. To investigate the relative stability of all metabolites, geometry optimizations were performed using Density Functional Theory (DFT)^48^, at the B3LYP level with the 6-311 + G(d,p) basis set^49,50^, via the Gaussian 16 program package^51^. The Polarizable Continuum Model (PCM) was employed to incorporate solvent effects, with methanol as the solvent medium^52–54^. All optimized structures exhibited C₁ symmetry, indicating no molecular symmetry constraints. Harmonic vibrational frequency calculations were carried out for each structure to verify that they correspond to true minima on the potential energy surface, as evidenced by the absence of imaginary frequencies.

### Statistics and reproducibility

All data were generated in three independent experiments (*n* = 3), and sometimes in four independent experiments as indicated in Supplementary File (Supplementary Fig. 6 and Table S3). All statistical details are mentioned in the methods section. Sample sizes were chosen based on standard sample sizes in the literature.

### Reporting summary

Further information on research design is available in the Nature Portfolio Reporting Summary linked to this article.

## Supplementary information

Transparent Peer Review file

SUPPLEMENTAL MATERIAL

Reporting summary

## Data Availability

All data are available in the main text or the supplementary materials. Source data for Table 1 can be found in the supplementary materials (Supplementary Fig. 6 and Table S3). Uncropped and unedited gels are depicted in the supplementary materials (Supplementary Fig. 7). All other data are available upon reasonable request from the corresponding authors. Gene sequences of *aprD* (PITCH_a420108), *aprE* (PITCH_a190075), and *apcA* (PITCH_a1910016) are available on uniport.
