# Supplementary material for: Breaking aromaticity in the anaerobic degradation pathway of phenanthrene comprises only ATP-independent type III aryl-CoA reductases
Source: Commun Biol. 2026 Jul 13;9:947. doi: 10.1038/s42003-026-10639-5 (PMC13365417; doi:10.1038/s42003-026-10639-5)
Supplement: Supplementary file 2 — SUPPLEMENTAL MATERIAL [file 42003_2026_10639_MOESM2_ESM.pdf]

Breaking aromaticity in the anaerobic degradation pathway of phenanthrene comprises only ATP-independent type III aryl-CoA reductases

Nadia A. Samak[1*], Marvin Häßler[2], Oliver J. Schmitz[2], Khadija Adjir[3], Rainer U. Meckenstock[1*]

[1] Environmental Microbiology and Biotechnology (EMB), Faculty of Chemistry, University of Duisburg-Essen, Universitätsstr. 5, 45141 Essen, Germany

[2] Applied Analytical Chemistry, Faculty of Chemistry, University of Duisburg-Essen, Universitätsstr. 5, 45141 Essen, Germany

[3] Laboratory of Thermodynamics and Molecular Modeling, Faculty of Chemistry, University of Sciences and Technology Houari Boumediene (USTHB), BP32 El Alia, 16111 Bab Ezzouar, Algiers, Algeria

[*] Corresponding author, E-mail:

nadia.samak@uni-due.de; Tel. +49 (0)201 183-7089

rainer.meckenstock@uni-due.de; Tel. +49 (0)201 183-6601; Fax +49 (0)201 183-6603

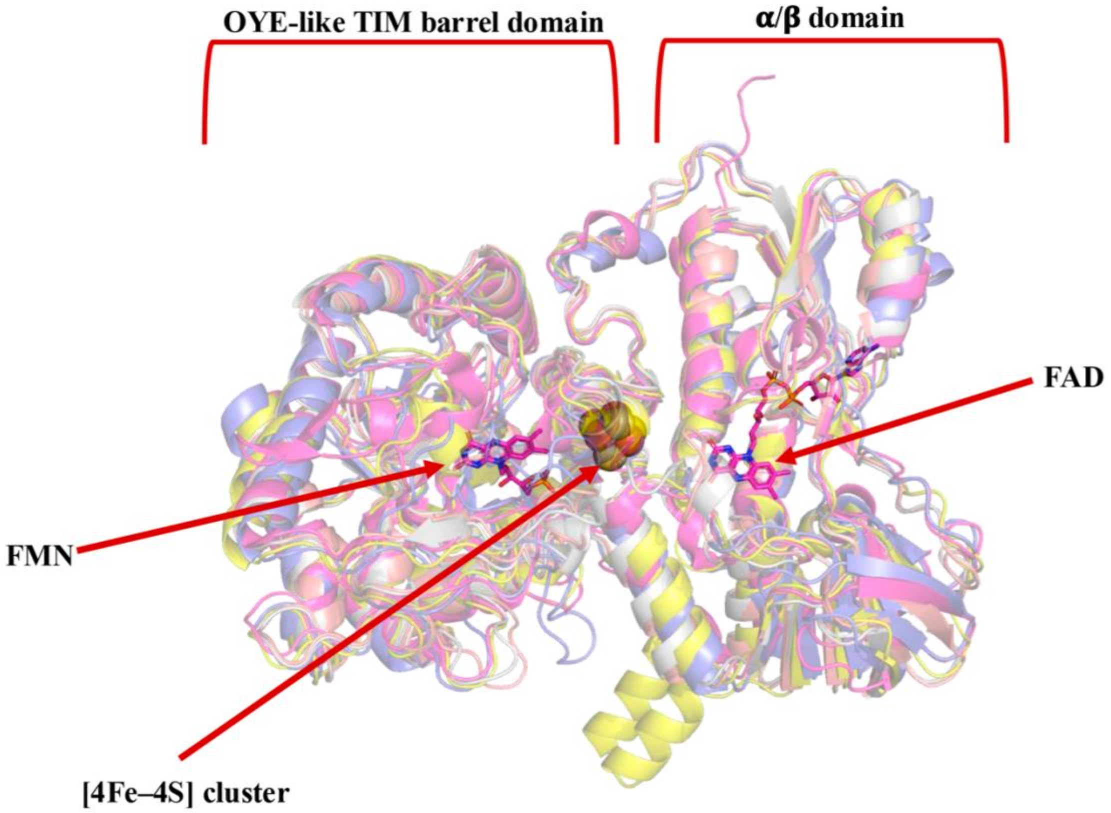

Fig. S1. 3D alignment using PyMol showing the fitting between the four enzymes of type III aryl-CoA reductases (AprB-E, pink, purple, grey, orange) and 2-naphthoyl-CoA reductase (yellow). The 3D structures were obtained using AlphaFold. To compare the possible reaction mechanisms of the four oxidoreductases with that of 2-naphthoyl-CoA reductase, the protein structures were modelled with the corresponding cofactors, such as FAD, FMN, and the [4Fe-4S] cluster. The cofactors were transplanted from existing crystal structures obtained from the PDB database for 2-naphthoyl-CoA reductase (6QKG). Each enzyme consists of one old-yellow enzyme-like TIM barrel domain and 2 α/β domains. The high sequence similarity and the 3d structure fitting of AprD and E with AprB, AprC, and 2-naphthoyl-CoA reductase recommend that the reduction mechanism of hexahydro-2-phenanthroyl-CoA and octahydro-2-phenanthroyl-CoA catalyzed by AprD and AprE should be similar to the reduction mechanism of 2-phenanthroyl-CoA (1) and dihydro-2-phenanthroyl-CoA (2) catalyzed by AprB and C, as well as to the reduction mechanism of 2-naphthoyl-CoA (3) catalyzed by 2-naphthoyl-CoA reductase.

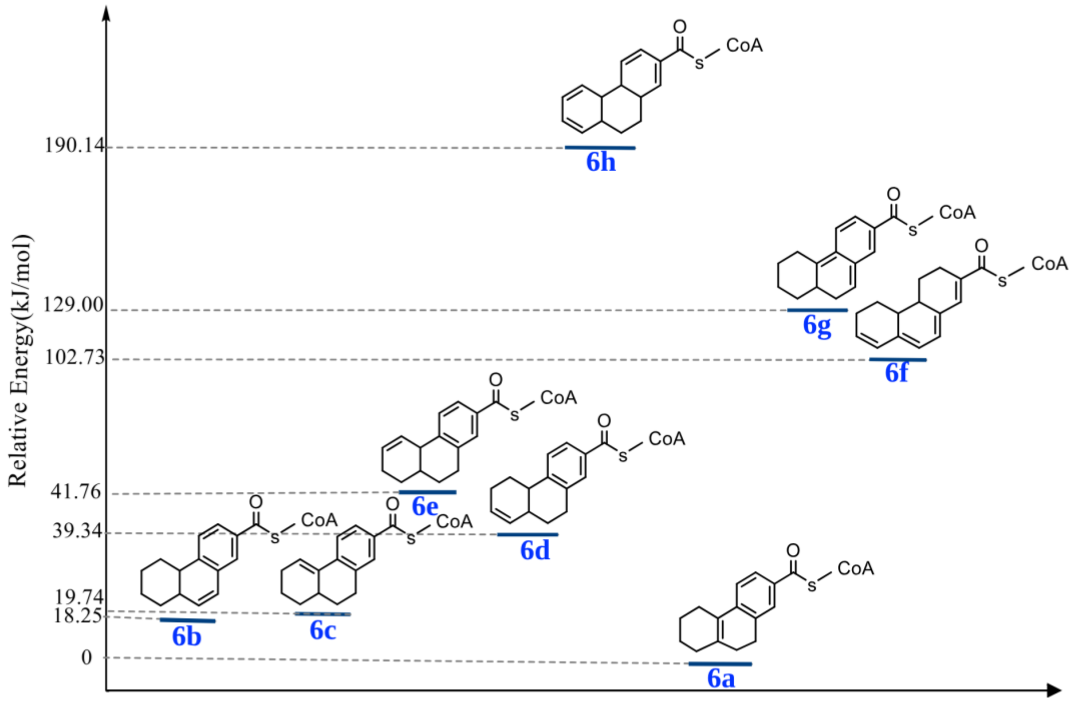

Fig. S2. Relative electronic energies [kJ mol^{-1}] of eight possible hexahydro-2-phenanthroyl-CoA **[6]** isomers calculated at the B3LYP/6-311+G(d,p) level of theory (2).

Table S1. Gibbs free energy changes (ΔG_{298} at standard conditions but 298 K and pressure 1 bar [kJ mol^{-1}]) for the reduction of each isomer of octahydro-2-phenanthroyl-CoA **[7]** to decahydro-2-phenanthroyl-CoA **[8]**

Isomer	ΔG_{298} (cal mol^{-1})	ΔG_{298} (kJ mol^{-1})
7a	-1851.41	-7.75
7b	-22985.38	-96.17
7c	-24160.08	-101.09
7d	-30146.68	-126.13
7e	-30860.16	-129.12
7f	-32734.83	-136.96
7g	-34167.71	-142.96
7h	-34261.05	-143.35
7i	-34151.81	-142.89
7j	-34809.31	-145.64
7k	-35837.54	-149.94
7m	-36659.78	-153.39
7n	-37636.78	-157.47
7o	-40405.57	-169.06
7p	-42286.7	-176.93

Table S2. Polymerase chain reaction primer sequences used for cloning *aprD*, *aprE*, and *apcA* genes. Primers are shown in the 5' to 3' direction.

Gene	Primer sequence
PITCH_a420108<br>(*aprD*)	F-primer: AGCGCGTCTCCAATGACGTCGAATTTTCCGAACTTAATGT<br>R-primer: AGCGCGTCTCCTCCCTGAATCGCCTCATAGATAAAGCG
PITCH_a190075<br>(*aprE*)	F-primer: AGCGCGTCTCCAATGGAGTTTCAGAACCTGTTTTCC<br>R-primer: AGCGCGTCTCCTCCCTCAGTCCAACTTCGAAGCC
PITCH_a1910016<br>(*apcA*)	F-primer: AGCGCGTCTCCAATGCTATTTACCCTGATAAGTGGGCT<br>R-primer: AGCGCGTCTCCTCCCATGGAGTACGAAAACATTATTTTG

Table S3. Determination of FMN and FAD content of AprD and AprE

	Exp. 1	Exp. 2	Exp. 3	Exp. 4	Mean	SD
**FMN content (AprD)**	0.4	0.6	1.1	X	0.7	0.4
**FAD content (AprD)**	0.4	0.9	1.45	X	0.9	0.6
**FMN content (AprE)**	0.3	0.8	1.3	0.2	0.6	0.5
**FAD content (AprE)**	0.3	0.7	0.8	1	0.7	0.29

a

CGTCTCCAATGACGTCGAATTTTCCGAACTTAATGTCGGTGTGCTATATTGGCAATG
TCGAGCTGAAAAACCGCATCATCACTGCGCCACTGTGGACTGGTTTTGCGGATCG
CGATGGTTCGGTAACCCCGCGTACGATTGCCTTTTATACCGAAAAAGCCCGTGGTG
GCTCTGGGCTGATTACCATCGAGTACTCCTATGTGGATCAGGTCGGCTCTAAATCT
GCCCATGGTCAGCTGGGTATCTATGATGATGAGTGCGTTCCGGGCTTTGCGATTCT
GGCGCAGACCATTCACGACCAAGGTGTGAAATGCGCAGTCCAGATTGCGCATGCT
GGAGCGATGAAATTTCTGCCGATTGCTCCGTGGCTTGGTCCGAGCGATGGGTTTCA
CGATCTCAGTCTGATGGGACCACTGCCTCCGTTGCCGATTACTGGCATGACCCGTG
AACAGATTTTCGAACAGATCAATGCTTTCGCATCAGCCGCAGAACGCGTGAAGAA
AGCGGGCTTTGATATGGTGGATATTCACGTTGCCCATGGATATCTGTTGACCGAATT
CTTAAGTACGCACGCTAATAAACGTGACGATGAGTATGGCGGGAGCTTAGAAAAC
CGCATGCGCTTCCCGATTGAGGTGGTTAGCGCAGTTCGGCAACGTGTTGGAGCCG
ACTTTCCGGTGACTGTCCGCATCAATGGCACGGATTATGCCCCTGATAGTCCTATCA
CCATCGACGAAGCGATCATCTTCGCGAAAAAGCTGGAAGAGGCGGGTGTAGATGC
GATCCACGTATCAGGCGGTACCGACATCTACCTGGACAAACTCGCGACGCCGACC
TACTTAAAACACGGATTCAACGTGTATTTGGCAGAAACGGTTAAGAAAAAAGCCG
GTGTCAAAGTCCCGATTATTGCTACCGGTGGTATCACCACACCGGCATTTGCGGAA
GAGATTCTTCGTGATGGTCGGGCAGATTTCATCGCCCTTGGGCGGCCTGCGTTAGC
AGACCCAAGCTGGGCGCGTAAAATCGAAGAAGGCCGCCCAGAGGACATCATCCC
GTGTATCCGGTGCAACGATGGGTGTCTGCGTCGCACAATTGGCTTTTCGCGTGCAA
CCAGCTGTGCCGTAAACCCGCGCATGGGCTTTGAAACGATTCGCGTGATTGCGCC
GCTGAAAGAGAAAAAACGCGTTGCCATCATTGGAGGTGGTCCAGGCGGGATGGA
AGCCGCACGGCTTGCTGCGATTCGTGGTCATGATGTGACCCTCTATGAGAAACGTC
GCTTAGGCGGGGCCTTGATTGAGGCGTCATGGGATACAGACCTTAAGCGTGATATT
CCGAATCTCATCGAGTACTACCAAACGCAGATGAAGAAACTGGATGTCAAGATCG
TGCGCAAAGAAGCAACCGTGAACTCTATTGCCGCTGGCGGGTTTGATGCCGTTAT
CGTTGCTAATGGCGCTATCTCGATCAAACTGGATATTCCAGGCATTGACAAGCTCC
ATGTGTATGAAGCGCTGGATGTGACAGGCGGGAAGGACAAAGACCTGGGCAATA
CCATTGTGGTCGTGGGTGGTGGGGTATTGGCCTGCGAAATTGCGCTGTCCCAAGC
GAAGAAAGGCAAAAAGGTTACGATTACGGCTCCGGAAGGATGTTATGCCGGCGA
ATACGAAATCGGCGGCGACAATTATCCGAATCGCTTAGCACTGATGGAACAGCTCA
AAGAGAACAAAGTGGGCATCAACCTGTGTCTGTTGCCGAAAGAAATCACCGATC
ATGGCATCATTAGCACTGACAAAGAAGGCAAAGAGCGCGAATTCAAAGCGGATAG
TATTGTTGTGTGCCGTGGTTTCGAACCTGATAAACGCCTGTCCAATGCATTGAAAG
GCAAAGTGAAACAGGTGCGTCCGATTGGTGATTGCGTACAAGCACGCTTTATCTAT
GAGGCGATTCATGA

b

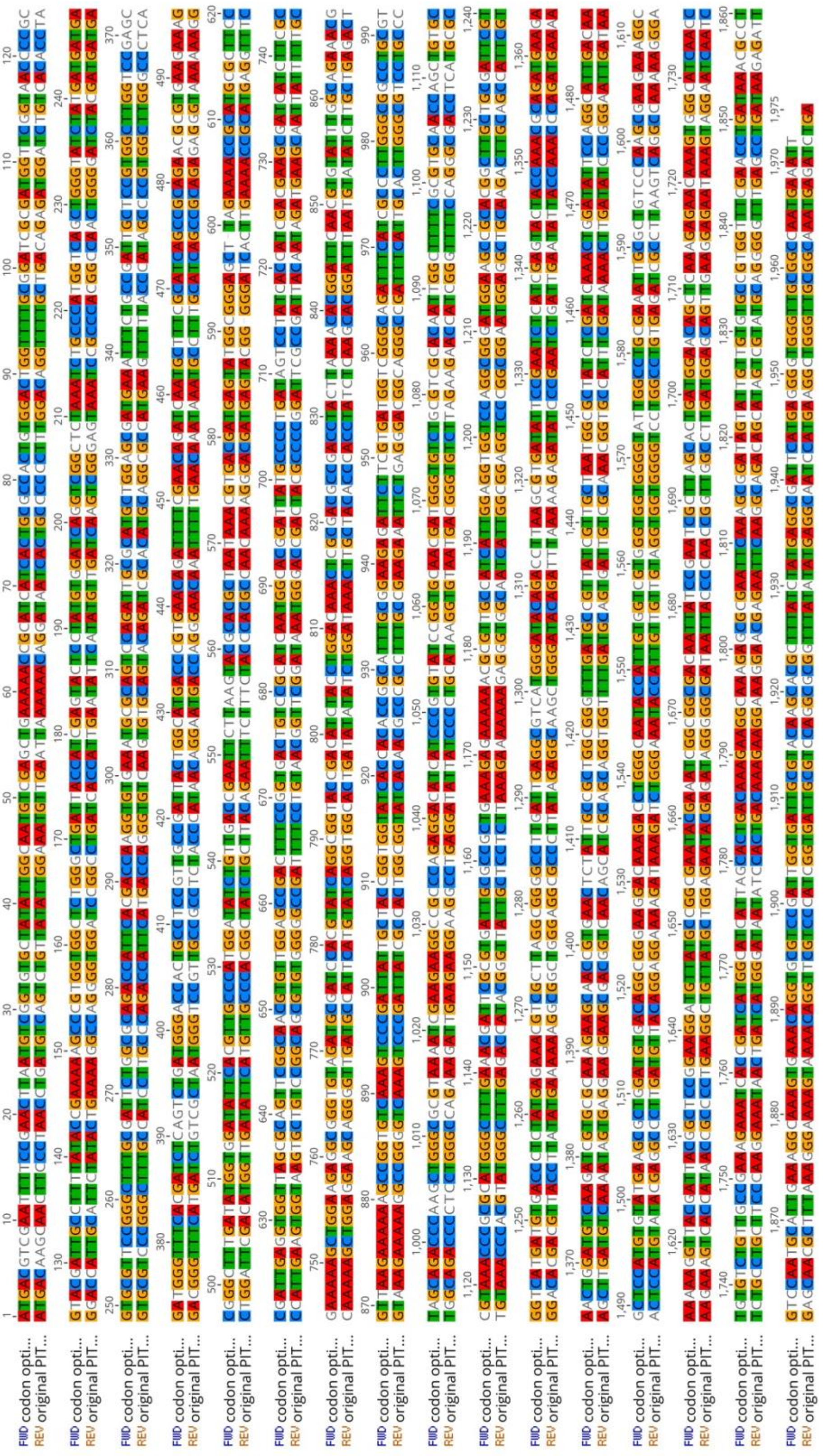

Fig. S3. Codon optimized gene sequence of PITCH_a420108 (*aprD,* 1954 bp) **(a)** and sequence alignment of the native and codon optimized *aprD* gene **(b)**.

a

ATGGAGTTTCAGAACCTGTTTTCCCCGATCAACATCAATGGGATGGCATTACGCAA
TCGCATTGTGATGACTGCAATGCACTTAGGCTATACCCCGCAAGGGGAAGTGACC
GACCGTTTGACGGAATTTTACCGGTTACGTGCTAAAGGCGGTGTGGGCTTGATTAT
CGTTGGTGGCTGTCGCTTTGATGATTATGGCGGTATGCCGTCGATGATTGGAATTAA
CGAGGATCGCTGCATTCCGGGTTTGAAACGGCTGACCGATACCGTCAAAGCGCAT
GGGGCCAAAATCGCCGCCCAGCTCTATCATGCAGGACGTTATACGCACTCCTCAAC
CATTGGCGGTAAAGCACCGTTTAGCGCTAGTGCGATCCGTAGCAAACTGACAGGG
GAAACACCGCGTGCGTTGGATACCCGTGAAATCCCACGCTTGCTCACTCTGTTTGC
GCAAGCTGCGGTTCGTGCGAAAGAATCGGGTTTTGACGCGGTTGAAATTTTGGGG
AGCGCAGGATACCTGATCAGCCAGTTTTTCTCTCCGCTGACGAATCAACGTCCTGA
CCTGTATGGCGGGTCCTGCGAAAATCGTATGCGCTTCGGTATTGAGGTGGTGGAAA
AAGTGCGGGATGCCGTAGGCCCGGATTATCCGTTAGTGATGCGTATTGCTGGTAAC
GACTTCATGGAAGGCGGGAACACCAACAAGGAAGCCAAAACCTTCGCGGCCGCG
TTAGAAGAAGCAGGGGTGGATCTGTTCAATGTAACGGGCGGTTGGCATGAGACTC
GCATTCCGCAGCTGACAATGAGCGTACCACACCGCGCGTTTGTGTACCTGGCAAA
AGGCATCAAAGATGCGGTCAAAGTTCCAGTATTGGCCTCCAATCGCATTAACGATC
CGGCCTTAGCCGAGGAGGTTTTGAAGAATCGGGAAGCAGATCTGGTCACCATGGC
CCGTGCGCTGATTGCGGATCCGGAATTGCCGGAGAAAGCACGTGGCAAAAAGAC
TCGCCTTATCTATCATTGCGTAGCCTGTAACCAGGGCTGCTTTGACTCGATCTTCAA
ATCGGAACCAGCCACTTGTCTGGTCAATCCTTGGGCAGGTCGCGAATGGGAGCTG
GAGAGCCGCTCTGCGCCTCGTCCGAAGAAAGTGCTCGTTATTGGAGGAGGCCCAG
CTGGCATGAAAGCGACGTGCGTTGCAGCAGGGCGTGGCCACAAAGTCACGCTCA
TCGAGAAAAAGGAGTATCTGGGCGGTCAACTGCTCCTGAACCGCAATATTCCTGG
GCGCCAGGAGCTGGTTGCTGCGGTGAGCGACCTCCTGAACAACCTGAAAGAACT
GAACGTGGAACTTATTCTTGGGAAAGAGGCGGATATCCACCTGGTGAAAGACATT
TCGCCTGATGCTGTCGTGATCGCCACGGGTGCAACTCCGATTTTGCCGAGTGTGCC
GGGCATCCATGGTCCAAATGTGCTGCATGCCTGGGATGTGCTTGCGGGGAAAGGC
AGTGCCGGAGACAAAGTGGTGATTGTTGGCGGCAATGCGGTTGGTCTTGAAACG
GCTCTCTATCTGGGTCGCCAAGGTACCCTTTCTCCGGAAGTCCTGCACTTTCTCAT
GATCAATCGCGCGGAAACCATTGACACCCTGTTTGCCTTGCTGGATCGTGGCGAC
AAAAAGGTGTCTGTAGTTGAGATGACCATGAAGCTTGGTCAGGATATTGGCGCGT
CAACGCGTTGGACCGTATTAGCGGAACTGGAACGCTTAGGCGTTACCATGCTGAA
AGGTTACCGGGCTGTAGAAGTGACACCGGAAGGCTTAATGACCGAGAAGGATGG
TAATGTCCAGCTGTTAAAAGCCGATACGGTGGTGATCGCAGTTGGTAGTCGCCCGG
AAAATGCGCTTGTCAATCAGGTGGAGTCACTGGTCACCGAGGCGTATACCATCGG
CGATGCAAAAGCCCCGCGTCATGCCCTGGATGCTATCCGCGAAGGCTTCGAAGTT
GGACTGATGA

b

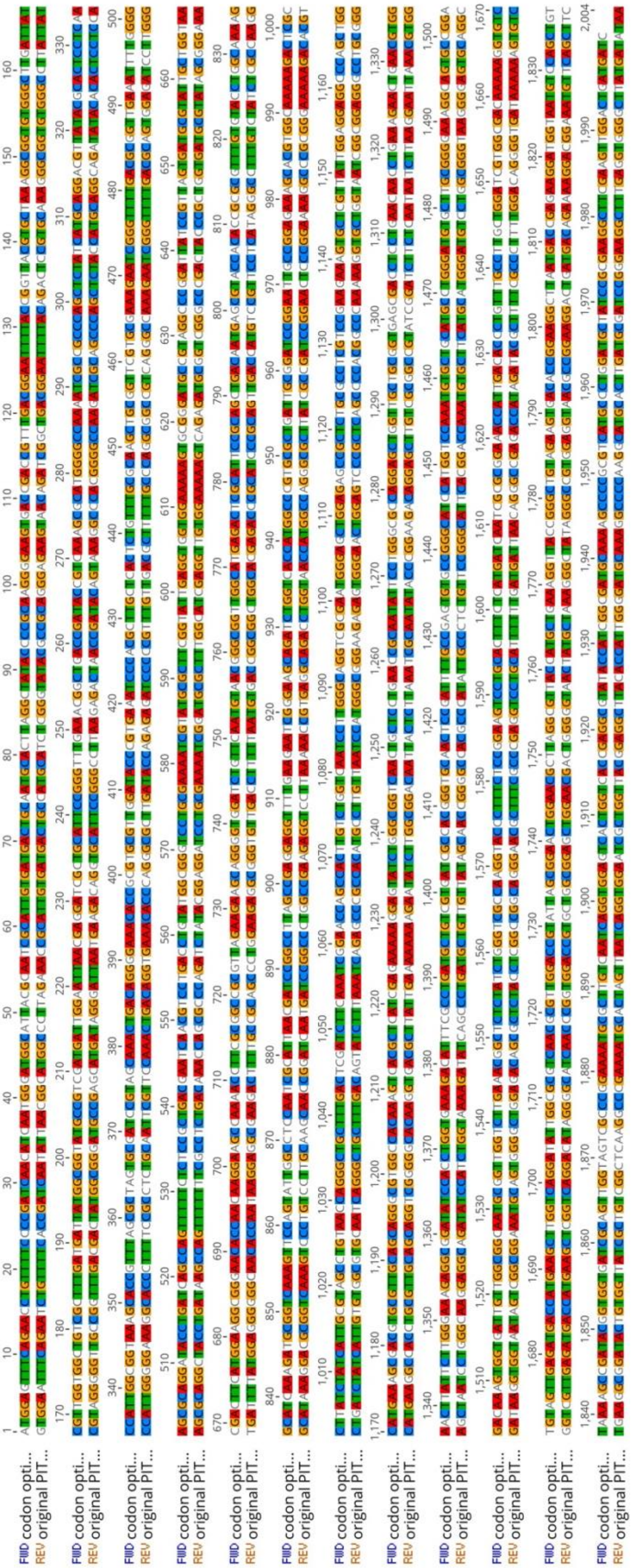

Fig. S4. Codon optimized gene sequence of PITCH_a190075 (*aprE*, 2024 bp) **(a)** and sequence alignment of the native and codon optimized *aprE* gene **(b)**.

CTATTTACCCTGATAAGTGGGCTTCCTTTTTTCGAGGAAAGCCGCAACACCTTCCTTCTTGTCATCGCTTGC
GAGAGCGAGATTTGCAGATAACGCCTCAAGCACAAGGCCGGTCTCCAGGTCAACTTCATCGCCGAATTTG
GTTGCTATCATTGCAAACTTTACGGCCAAGGGTGCTTTTGTGGCGATCTTCTTTGCGAGCTTCATCGCAAC
GTCCATAAGCTCTTCAGGCTTGGCCACCTGATTTGCAAGCCCCAAGGCCAGGGCCTTGTTGGCATCGAGG
TTTTCACCTGTCAGCATGGCCCACAGGGCCTGACTCCTTCCGATGATCCGCGGGAGTCTCTGTGTGCCGC
CATAGCCGGGGATAACACCCAGCCCAAGCTCAGGAAGACCGAACAGTGCGTTTTCCGAAGCAATCCTGA
AGGTGCAGCCCATTGCAAGCTCGCAACCTCCGCCCAAGGCCATTCCGTTTACCGCTGCAATTGTTGGAAC
ATTGAGTGCCTCGAGGTCTCTGAAAATGCCTGCGCCCCTTCTGATGAAGTCATAACTTTCCTTCAGGCCCA
GCCCCGTAAACTCGGATATATCAGCACCCGCCACAAAGGCCTTGTCGCCGGCGCCTGTAATAATCATCGCC
CTGATGGAATCATCGCCCTTTACCGTGGCTATAGCGTCTTTTATCTCGTCCATAGTGCGGAAATTAAGGGCG
TTCATCTTTTTTGGCCTGTTTACCGTCAGGACCGCAATGCCTTCCTTGACCTCAAAAATAATGTTTTCGTAC
TCCAT

Fig. S5. Gene sequences of PITCH_a1910016 (*apcA*, 783 bp) encoding enoyl-CoA hydratase.

a **hexahydro-2-phenanthroyl-CoA conversion to octahydro-2-phenanthroyl-CoA**

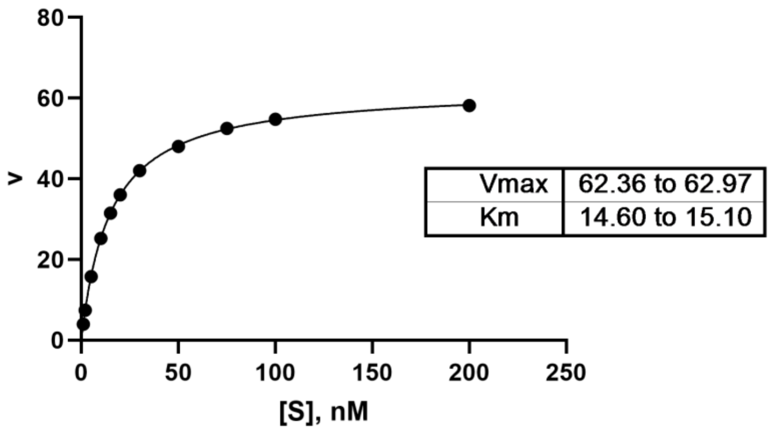

	Exp. 1	Exp. 2	Exp. 3	Mean	SD
**K_M** (nM)	13.2	19.24	15.12	15.1	4.2
**Specific activity** (nmol min^{-1} mg^{-1})	16.6	10.67	12.66	12.66	4.1

b octahydro-2-phenanthroyl-CoA conversion to decahydro-2-phenanthroyl-CoA

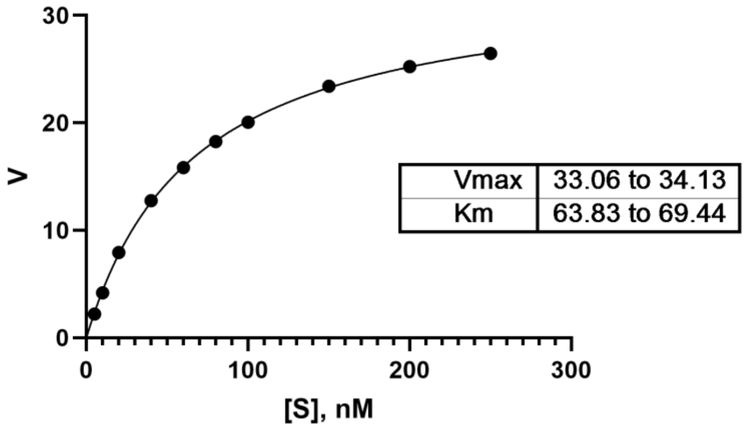

	Exp. 1	Exp. 2	Exp. 3	Mean	SD
**K_M** (nM)	63.9	77.1	40.9	63.9	9.3
**Specific activity** (nmol min^{-1} mg^{-1})	6.3	9.7	5.5	6.3	2.3

c decahydro-2-phenanthroyl-CoA conversion to β-hydroxydodecahydro-2-phenanthroyl-CoA

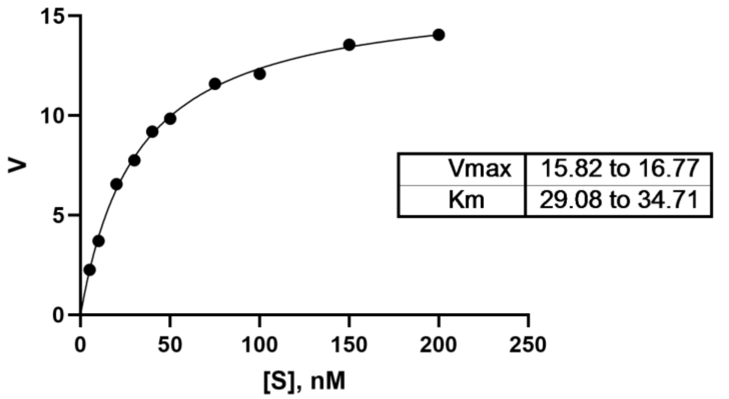

	Exp. 1	Exp. 2	Exp. 3	Mean	SD
**K_M** (nM)	31.51	36.3	28.1	31.5	4.1
**Specific activity** (nmol min^{-1} mg^{-1})	3.22	4.1	2.4	3.22	0.9

Fig. S6. Kinetic properties calculations of **(a)** hexahydro-2-phenanthroyl-CoA reductase (AprD), **(b)** octahydro-2-phenanthroyl-CoA reductase (AprE), and **(c)** enoyl-CoA hydratase (ApcA)

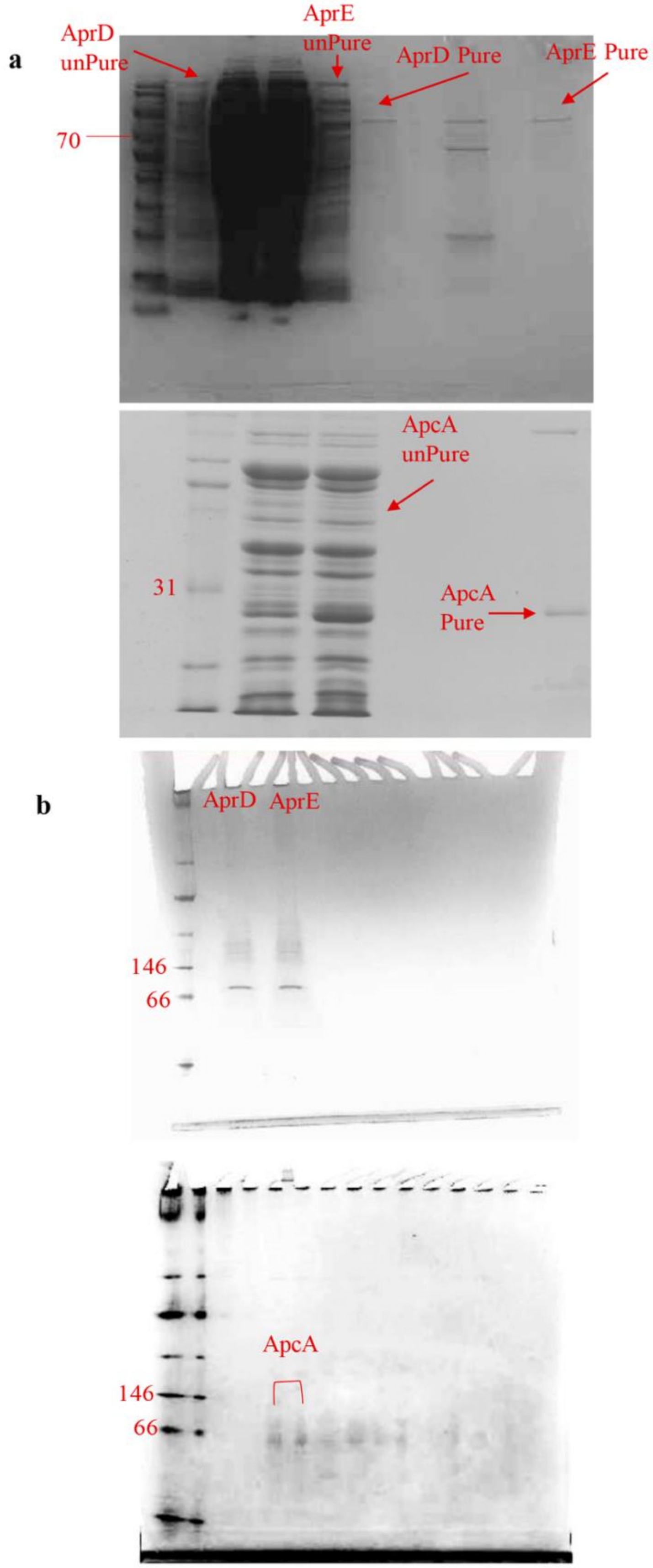

Fig. S7. Uncropped and unedited gels of **Fig. 2.**

References

1. Kaplieva-Dudek I, Samak NA, Bormann J, Kaschani F, Kaiser M, Meckenstock RU. 2024. Characterization of 2-phenanthroate: CoA ligase from the sulfate-reducing, phenanthrene-degrading enrichment culture TRIP. Appl Environ Microbiol doi:10.1128/aem.01296-24:e01296-24.
2. Samak NA, Adjir K, Götz F, Surmeneva A, Fax J, Haberhauer G, Meckenstock RU. 2026. The ATP-independent dihydro-2-phenanthroyl-CoA reductase AprC catalyzes two consecutive two-electron reduction steps of dihydro-2-phenanthroyl-CoA to hexahydro-2-phenanthroyl-CoA in anaerobic phenanthrene degradation. Appl Environ Microbiol 0:e02248-25.
3. Eberlein C, Estelmann S, Seifert J, Von Bergen M, Müller M, Meckenstock RU, Boll M. 2013. Identification and characterization of 2-naphthoyl-coenzyme A reductase, the prototype of a novel class of dearomatizing reductases. Mol Microbiol 88:1032-1039.
