# Supplementary material for: Breaking aromaticity in the anaerobic degradation pathway of phenanthrene comprises only ATP-independent type III aryl-CoA reductases
Source: Commun Biol. 2026 Jul 13;9:947. doi: 10.1038/s42003-026-10639-5 (PMC13365417; doi:10.1038/s42003-026-10639-5)
Supplement: Supplementary file 1 — Transparent Peer Review file [file 42003_2026_10639_MOESM1_ESM.pdf]

nature portfolio

Peer Review File

Breaking aromaticity in the anaerobic degradation pathway of phenanthrene comprises only ATP-independent type III aryl-CoA reductases

Corresponding Author: Dr Nadia Samak

This file contains all reviewer reports in order by version, followed by all author rebuttals in order by version.

Version 0:

Reviewer comments:

Reviewer #1

(Remarks to the Author)
The paper by Samak et al investigates the function of two members of the aryl-CoA reductase family, AprD and AprE that catalyze flavin-mediated reduction of intermediates in the anaerobic phenanthrene pathway. Importantly, the reactions function independently of ATP, yet are hypothesized to catalyze dearomatization of the benzene ring. This mechanism contrasts that of the naphthalene degradation pathway, where the benzene ring of tetrahydro-2-naphthoyl-CoA is reduced by an ATP-dependent type I aryl-CoA reductase.
Overall, the paper provides insight into the anaerobic degradation of PAH, revealing that dearomatization does not necessarily require ATP.

Major Comments
Line 236 and line 225. Why is 1 mM NADH added to the reaction mixtures?
Line 287. It is not clear how the authors were able to obtain pure quantities of hexahydro2-phenanthroyl-CoA, octahydro-2-phenanthroyl-CoA and decahydro-2-phenanthroyl-CoA. Figures 4-6 indicate that very little product was formed. How were these isolated from the starting material and quantified?
Line 278. It is not clear from the Methods and Materials section how the macroscopic kinetic parameters (kcat/specific activity and Km) were obtained for all three characterized enzymes. AprD, AprD and ApcA0. Data should be presented for the steady-state experiments. It is unclear how Km values in the nM range were obtained when the substrate concentrations varied from 1 -100 uM or 1 to 50 uM.
Lines 295 to 511 are missing from the manuscript.
Line 607. Please explain the unusually large errors associated with the FAD and FMN content. How were these SD values determined?
Table 2. How was NADH 5% - maximal activity determined?
Figure 1. If there is sequence similarity among ArpB, C, D, and E, it would make more sense, given the similarities in active-site geometry, that double bonds on the periphery of the rings would be reduced.
Minor comments
Line 47, remove “but water”
Lines 54, 769, change to “monoaromatic”
Line 60, change to “This constitutes a new degradation strategy that is distinct from the anaerobic degradation of…”
Line 132: Rewrite the sentence beginning with “In this study…” It is not coherent in its present form.
Line 220. Remove “therefore” at the end of the sentence
Line 223 and elsewhere. Change “enzymatically accumulated” to “enzymatically formed”
Line 224. Remove the second “reduction”
Line 603. Small case “flavin mononucleotide”
Line 606. Small case “flavin”
Line 612 change to “cofactors”
Line 807 remove “kind of”
Line 812. Perhaps end the sentence with “ATP hydrolysis”
Line 818. Split the sentence beginning with “Both types of” into two sentences.
Line 835: change to “to be asymmetric”

Line 883. Change to “It also catalyzes the”
Line 884. Remove the word “however”
Line 889. Change to “non-aromatic”

Reviewer #2

(Remarks to the Author)
General comments
The study describes the last enzymatic steps in anaerobic bacterial phenanthrene degradation prior to ring-opening, comprising two subsequent two-electron reductions and an addition of water to the resulting cyclic diene system. Most important, it could be demonstrated that also the final two-electron reduction step from octahydro-2-phenanthroyl-CoA to decahydro-2-phenanthroyl-CoA yielding a fully dearomatized system is catalyzed by an ATP-independent type III aryl-CoA reductase. This is somewhat surprising, as related reactions in degradation pathways of benzene and naphthalene are catalyzed by ATP-dependent type I aryl-CoA reductases where ≥2 ATP are hydrolyzed per two-electron transfer to overcome the resonance energy of the aromatic system. Alternatively, ATP-independent type II aryl-CoA reductases are employed for the dearomatizing step, overcoming the resonance energy by energy input via an electron-bifurcating mechanism. In contrast, the “simpler” type III reductase-catalyzed reaction seem not to include any substantial energy input reaction mechanism to overcome the resonance energy.
Overall, the work is convincing. Unfortunately, lines L303 to L511 are missing in the manuscript (of the Materials and Methods part). The passages dealing with activation/resonance energies might be revised to some extent, as activation energies obviously differ (L822) in reducing aromatic 1-, 2- and 3-ring systems (see specific points). In addition, I found the low KM values (in the 30 to 150 nM range, Table 2) quite puzzling, as only substrate concentrations ≥1000 nM were tested to determine the kinetic parameters (L288). Maybe, you can provide corresponding kinetic plots and non-linear regression analysis in the supplement. Besides, I have several minor points that might be addressed to improve the manuscript (see below).

Specific points
L31 “…two-electron reductions steps…” change “reductions” to “reduction” (see L62).
L34 “Km” I would prefer “KM” with lowercase “M” (see Table 2).
L45 “pH value of 7.5” Ok, and for the other reactions?
L54 “Anaerobic degradation of mono aromatic compounds and PAHs such as phenanthrene…” Abbreviation “PAH” not introduced (see L74).
L56 “…that after phenanthrene is activated to a CoA-ester…” change “CoA-ester” to “CoA-thioester”.
L69, KEYWORDS: “culture TRIP 1” Maybe, you should change to “enrichment culture TRIP 1” (see L85).
L78 “…stable PAHs and to brake the aromaticity…” Looks unusual to me. Maybe, change “brake” to “break”.
L81 “…is well studied to a certain extent…” Well or to a certain extent? It is always studied to a certain extent. I would omit the “certain extent”.
L82 “…biodegradation of phenanthrene and larger-PAHs [1]…”, L84 “…degradation pathway of phenanthrene [1]…”, L86 “…Trinidad and Tobago with phenanthrene [1]…” etc. Obviously, “[1]” refers to the compound phenanthrene (see also numbering of compounds in Fig. 1 and caption L152). But after introduction, you can just give the number and omit the compound name in the text. Otherwise we do not need the numbers. See also the numbering of the metabolite compounds (L92, 93…).
L86 “…the Pitch Lake in Trinidad and Tobago…” I wonder whether there is only one (“the”) pitch lake in Trinidad and Tobago. See ref. 10 (https://doi.org/10.1111/1462-2920.14335) = “at the Pitch Lake in La Brea, Trinidad”.
L94 “…the 2-naphthoate:CoA ligase (apaC) is located…” I think you mean the 2-phenanthroate:CoA ligase (of ref. 8).
L94 “…putative carboxylase gene clusters (apaA,B,F,G, I, and J)…” Well, the term gene cluster seems not to be well defined. But I would use here (= at least for the cluster of all apa genes) singular and not plural. See also L128 “constituting a gene cluster”.
L100 “genome of culture TRIP 1” As it is an enrichment culture but not a bacterial isolate (??), I would rather refer to the “metagenome”. Or is it the MAG of Desulfatiglans TRIP_1? See also L114.
L114 “In the genome of the anaerobic phenanthrene degrader TRIP 1”? Does “degrader” refer to an isolate?
L116 “…the neighborhood of the type III aryl-CoA reductase genes…” Well, the “neighborhood” is not essential. Are there genes for type I reductases in the genome/metagenome/MAG? And what about genes encoding ATP-independent type II aryl-CoA reductases? The latter seem also be relevant for sulfate-reducing bacteria (see Environ Microbiol 2019 Nov;21(11):4241-4252. doi: 10.1111/1462-2920.14784).
L117 to 121 “This is a very important problem because…” Well, that the Gibbs free energy change of the complete process (= multiple reactions) is negative is not sufficient. (And “delta G” is not an appropriate writing.) For each single reaction step (i.e., for each abiotic or enzymatically catalyzed reaction), the Gibbs free energy change must be negative = each step must be exergonic. Otherwise, the reaction(s) would not take place. Ok, obviously, the Gibbs free energy changes are “more or less in the same range for the different reduction steps of mono-aromatic compounds and PAHs”. But is ref. 12 really appropriate for this statement? Does ref. 12 provide relevant values (of 1-, 2-, 3-ring systems) for the Gibbs free energy changes? And ref. 15 only deals with activation energy required for the dearomatization of the 1-ring system. This can obviously not simply be applied for other systems (see also your conclusion in L822). Are there any (theoretical) values available that describe activation energies for the different reduction steps? At least, the activation energies for the initial reduction steps of the 2- and 3-ring systems are lower than for the dearomatization of the 1-ring system. Consequently, type III reductases can catalyze the reactions, somehow “overcoming the resonance energy of the aromatic ring-system” (L96).
L124 “…PAHs with three (e.g. phenanthrene) or more rings can work if they do not possess…” Incomplete sentence. Specify “they”.

Fig. 1: Compound 3 “2- phenanthroyl-CoA” Remove spacebar. Generally, the “S” of the S-CoA is not in the same font size as the “O” or the “CoA”. Why?
Fig. 1, caption. “(a) Graphical presentation of the gene clusters encoding enzymes responsible for anaerobic phenanthrene activation (apaA-apaJ),…” In L94, you refer to the carboxylase genes as putative, indicating that the corresponding enzyme activity has not yet been established/identified. So, what is true? Responsible or putative responsible?
L265 “…the reaction was performed with or without 50 µM ATP.” Why only 50 µM? Compared to the concentration of the electron donor components (added in the mM range) this is quite low.
L294 “M. Braun…” Check formatting. Do you mean M. Braun Inertgas-Systeme GmbH?
L303 to L511 are missing in the manuscript!
Results part: Do not repeat all the experimental details and what is already described in the Materials and Methods. For example, L577 “purified separately using a Strep-Tactin XT 4Flow gravity-flow column” or L645 “…the enzymatic reaction was stopped by adding a double volume of methanol. The methanol was evaporated, and the aqueous solution was freeze-dried to prepare a substrate stock of octahydro-2-phenanthroyl-CoA”.
Fig. 2: The gel image for ApcA in (b) looks somewhat distorted. I can hardly see the bands. Please check.
L605 etc. Do not simply repeat all the values (with SD) in the text that are nicely provided in Table 2.
L843 “If octahydro-2-phenanthroyl-CoA reductase AprD produces a non-aromatic isomer with conjugated double…”. Yes, but then the AprD-catalyzed reaction would be challenged by (possibly) high activation energy to overcome the aromatic system.
Fig. 8: I wonder whether these calculations are also available for the naphthalene system. Could it explain the need for type I reductase employment and ATP-consumption in the last reduction step to yield the fully dearomatized system in the naphthalene pathway?

Reviewer #3

(Remarks to the Author)
This is a very nice study that successfully illuminates some new biochemistry in the anaerobic degradation of hydrocarbons. Enzyme candidates have been identified, heterologously expressed and purified, and a pathway for phenanthrene degradation reconstituted in vitro. The paper is very well written overall with few typographical errors although the figures could be clearer. The data appear to be in order and the methods are described with sufficient clarity to permit reproduction. Some minor comments:

Abstract Line 27 – why are these enzymes specifically described as ‘ATP-independent’; most enzymes are?

Line 78 ‘break’

Fig 1 – harmonise size of S atom labels in these structures – not clear why they are smaller than others

Methods – min, not minutes; Michaelis ‘K’ is italic

Line 598 – use ‘OYE’ early in the manuscript – this is an accepted abbreviation and can then be used throughout

Line 612 – change ‘optically active’ as this may inadvertently suggest chirality and the preponderance of one enantiomer

Figure 4 Page 22 – legibility of text and structures is an issue here – please reconsider format; also Figure 5

Line 742 remove ‘is real’

Figure 8 – need to rethink this; again for legibility and the proper representation of chemical structures.

Reviewer #4

(Remarks to the Author)
It’s been an exciting year for the field of anaerobic hydrocarbon biodegradation. Several new papers and preprints have been published on the characterization of associated enzymes and metabolic reactions, especially for PAHs. Drs. Samak, Meckenstock, and their team have published a series of papers on the characterization, heterologous expression, and activity of enzymes involved in phenanthrene biodegradation under sulfate-reducing conditions, specifically 2-phenanthroate:CoA ligase (10.1128/aem.01296-24), phenanthroyl-CoA reductase (10.1128/aem.00166-25), and dihydro-2-phenthanthroyl-CoA reductase (10.1128/aem.02248-25). This fourth paper continues these efforts and describes three new enzymes that participate in ring reduction (AprD, AprE) and hydrolysis steps (ApcA) of anaerobic phenanthrene degradation by a Desulfatiglans-affiliated bacterium in the sulfate-reducing enrichment culture TRIP 1.

Prior to reading this latest paper, I was aware that the Meckenstock lab had enriched for a phenanthrene-degrading enrichment culture (Himmelberg et al., 2018; doi:10.1111/1462-2920.14335) but was not well versed in their more recent enzyme characterization efforts. Figure 1 and Lines 84-125 provide a helpful overview of the aforementioned papers and their major discoveries. However, I found that some concepts central to this paper were glossed over. For example, what exactly are type III aryl-CoA reductases, when were they first described, and how do they differ from type I and type II aryl-CoA reductases? What is the old yellow enzyme family? Are all old yellow enzymes type III aryl-CoA reductases? It was

only after I carefully read through Samak et al.'s previous papers that I found answers to some but not all of these questions. While each paper builds on the last, which supports continuity, this approach risks leaving key concepts insufficiently defined for readers who are not already familiar with the series. The authors should be mindful of this, and be sure to introduce all relevant concepts, organisms, and enzymes in sufficient detail when they first appear in each manuscript.

The experiments and data presented in this study are technically sound, and the work is overall very impressive. One Materials and Methods section (starting on line 291) appears to be missing a substantial amount of text (perhaps due to a copy/paste error) and should be corrected. I do have a few questions regarding this study's enzyme activity assays. The CoA-ester substrates used for enzyme activity assays were synthesized enzymatically. Based on the t = 0 min data presented in Figures 4 and 5, it appears that the target CoA-ester substrates were not (or could not be) purified prior to use (i.e., residual 2-phenanthroyl-CoA, 7,8-dihydro-2-phenanthroyl-CoA, etc., remain present). Please confirm whether this is the case. If so, could these residual compounds have influenced the apparent rates of AprD, AprE, and ApcA? Additionally, what reference standard(s) or LC-MS calibration approaches were used for quantification of each synthesized CoA-ester?

Well done, authors! Some specific line comments below.

L84: "In this study, we uncover the upper anaerobic degradation pathway of phenanthrene …" This is somewhatt misleading. This paper only describes three steps in this pathway.

L87-88: "The dominant organism of culture TRIP 1 belongs to the genus Desulfatiglans within the family Desulfobacteraceae". How many organisms are present in TRIP 1, and what proportion of the community is comprised of Desulfatiglans? Himmelberg et al. (2018; doi:10.1111/1462-2920.14335) describes four organisms based on metagenomic sequencing and MAG assembly (Desulfobacteraceae/Desulfatiglans, Geobacter, Paludibacter, and a Spirochaete.) Has this community structure changed since ~2018, and how is it being monitored? As an aside, I checked the NCBI entry for the TRIP 1 metagenome and assembly data (PRJEB27924) and found that the Desulfatiglans genome assembly was suppressed (annotation fails MAG completeness check; see https://www.ncbi.nlm.nih.gov/datasets/genome/GCA_900498135.1/). Please review. Has a more recent Desulfatiglans genome been sequenced and assembled?

L89-107: The authors should clarify that the phenanthrene degradation pathway overviewed in this paragraph is specific to Desulfatiglans in TRIP 1 and is not necessarily a universal phenanthrene degradation pathway. We do not yet know the full breadth of microbes and biochemical mechanisms capable of catalyzing PAH degradation. Anaerobic benzene biodegradation, for example, can be initiated by several activation mechnanisms including but limited to carboxylation.

L104: What is "two times two-electron reduction"?

L112-113: Please consider introducing Azoarcus, Deltaproteobacteria N47 and strain NaphS2 as model organisms used to study anaerobic benzene and PAH biodegradation, as well as all three types of aryl-CoA reductase enzyme families, to make the manuscript more accessible to readers who are not subject-matter experts.

L129: What does thn stand for? The authors may also wish to define the abbreviations for apr and apc in the main text, as they are easy to miss in Figure 1.

Figure 1: The authors may wish to consider adding citations to specific papers under each characterized reaction step so that readers can locate relevant studies more easily. In the figure caption, please explain why is there a red X through 9,10-dihydro-2-phenanthroyl-CoA (4c).

L195: Why were these volumes chosen? Please provide additional detail.

L214: This is a very old reference (from 1963). Is there a more recent protocol that can be cited, particularly one that reflects updates to the original procedure?

L580: Any insights into the identity of this impurity?

L583: Should be ApcA (capital A).

L592: Should be kDa (lowercase k). Please review the text carefully for similar formatting errors.

Table 2: Consider specifying the number of replicates performed per assay and what the error bars represent.

L657-660: I was surprised to read that AprD and AprE do not appear to be oxygen sensitive. How does this compare to other characterized "old yellow" and type III aryl-CoA reductases?

L665-666: Are these data presented anywhere? It might be worthwhile to include them as Supplemental Information, including electron donor concentrations. Negative results can be informative for other research groups working on enzyme characterization.

L764-766: "…which is performed by either a fumarate addition to methyl groups or a carboxylation of the aromatic ring in the case of non-substituted compounds such as benzene and PAHs." Fumarate addition and carboxylation are not the only

known biochemical mechanisms of anaerobic hydrocarbon activation.

L800-802: Now that several type III aryl-CoA reductases have been characterized, it would be worthwhile to construct a phylogenetic tree of this enzyme family. Consider including uncharacterized homologs from hydrocarbon-degrading isolates and enrichment cultures beyond NaphS2 and N47. This would be very helpful for understanding the substrate diversity and evolutionary diversity of type III aryl-CoA reductases. Bootstrap support values should be included.

L829: "… is supported by previous results …" Please provide an appropriate citation.

L867-875: Consider preparing a phylogenetic tree for ApcA and representative enzymes within the crotonase superfamily. Does ApcA form a distinct clade or does it cluster with known enzyme groups? The reported 42-60% gene sequence identity is very high - did you mean amino acid identity?

Version 1:

Reviewer comments:

Reviewer #1

(Remarks to the Author)
The authors have addressed my concerns.

Reviewer #2

(Remarks to the Author)
I do appreciate that most of my comments have been addressed appropriately in the revision of the manuscript. However, there are still things to do. It is good that you have corrected several (serious) typos (KM and SD values, concentration units of substrates used in assays). But you have to correct them throughout (see L34). And sometimes, the revision did not improve the manuscript (e.g. L149, see comment below). Moreover, to be as consistent and correct as possible in a manuscript is not just for fun. It is important for clearness and will help to prevent propagation of errors in future studies. You should also not state in the rebuttal that something was corrected when it is not (e.g. see comment below on L116).

L34 "12.7 and 6.3 nmol min-1 mg-1 and KM values of 150.7 and 63.9 nM, respectively." In the rebuttal, you indicated a typo ("The apparent KM value of AprD was typo mistake it should be 15.07 (15.1) instead of 150.7,…"). And in Table 2 it is corrected. But you have to correct it throughout!

L92 "old-yellow enzyme family (OYE)" change to "old-yellow enzyme (OYE) family" (see L40, L58 etc.).

L116 "The gene encoding the 2-naphthoate:CoA ligase (apaC) (25) is located in the vicinity of the putative carboxylase gene cluster…" In the rebuttal, you stated that it was corrected. But it is still "2-naphthoate:CoA ligase", although the context and ref. 25 indicate that it should be the "2-phenanthroate: CoA ligase". See L115 (ApaC = phenanthroate:CoA ligase).

L144 "This is a very important problem…" I think the passage is ok. Just a small reply on your rebuttal comment "Some exergonic reactions can pull endergonic reactions such as in e.g. syntrophic methanogenesis." Yes, the effective concentration (activity) of hydrogen is kept very low in this synthrophy, resulting in the "pull" from endergonic to exergonic. But, of course, endergonic refers here to standard conditions and also to a wide range of other hydrogen activity values. At very low hydrogen activity (at real conditions and due to the very efficient hydrogen consumption in the synthrophic system), however, the reaction is exergonic = it can take place. Standard conditions or other reference conditions (e.g. at neutral pH) are useful to compare different reaction steps in a pathway and to identify thermodynamic bottlenecks. But we have to keep in mind that in the real world, the Gibbs free energy change (of each single reaction) depends on the real conditions (temperature, activity values of substrates and products). So, when giving values for the Gibbs free energy change, you should indicate the conditions (e.g. standard conditions corrected for pH = 7). See also L652 and Fig. 9.

L149 "Hence, it is unclear how type III aryl-CoA reductases can reduce the aromatic ring systems of larger PAHs with three (e.g. phenanthrene) or more rings without additional energy input from ATP-hydrolysis, if these larger PAHs do not use type I aryl-CoA reductases.
I am sorry, but this is not better than before. PAHs "do not use" something. The original sentence was: "Hence, it is unclear how the reduction of the aromatic ring systems of larger PAHs with three (e.g. phenanthrene) or more rings can work if they do not possess type I aryl-CoA reductases." I think the "they" should refer to the degrading system = the microorganisms. They can possess or lack the relevant enzymes.

L248 "Enzymatic synthesis of CoA-ester substrates"
L306 "phenanthroyl-CoA ester [8]"
L307 "decahydro-2-phenanthroyl-CoA ester"
L310 "decahydro-2-phenanthroyl-CoA ester"
L315 "hydroxydodecahydro-2-phenanthroyl-CoA ester"
L442 "of 2-phenanthroyl-CoA ester"
Change "ester" to "thioester" (see L56) or omit "ester" where it is not needed (e.g. L315).

L330 “Vmax” Use consistent writing throughout (see L325 = italic and subscript).

L440 “…showed an absorption at 280 nm, …” Maybe, change to “…showed only an absorption peak at 280 nm, …”. Of course, also the flavin-containing enzymes show substantial absorption at 280 nm, but ApcA lacks the peaks around 375 and 450 nm.

Fig. 6, L558 “β-hydroxydodecahydro-2-phenanthroyl-CoA [9]”. Sorry for not commenting in my first review. But looking at Fig. 6 again, I am not sure with the structure of the ApcA reaction product = compound [9]. In Fig. 1, you indicate two isomers for [9], but here only one. See also section title (“…adds a water molecule to one of the double bonds…”). Could LC-MS confirm the isomer depicted in Fig. 6? Or is it possible that the other or that both isomers were formed?

L670 “SP3 hybridized” change to “sp3 hybridized” with the “3” as superscript.

L648, L652, L684, L685, Fig. 8, L692 “kJ/mol” I would prefer “kJ mol-1” with “-1” as superscript. See L678.

Fig. 9, L695 “Gibbs free energies” I would prefer “Gibbs free energy changes”. And, as always, you have to give the units (I guess the values are in kJ mol-1).

Reviewer #4

(Remarks to the Author)
It was a pleasure to revisit this manuscript. My questions and comments were appropriately addressed by the authors; thank you. I look forward to learning more about the phylogeny of type III aryl-CoA reductase and ApcA in your forthcoming manuscripts.

A few minor corrections:
L84 - It's odd to start citing your chemical structures from [13] (benzoyl-CoA), which is first shown in Figure 9. Consider removing [13] here to avoid a lot of reformatting.
L109 - 1.3 is missing a %; Desulfarculus should be italicized.
L121; 141 - are you referring to amino acid or nucleotide sequence identities? This should always be specified.
L164 - apcA should be lowercase and italicized.
L243 - SD should be written out in full.
L330 - Vmax formatting should match the rest of the main text.
L496; 685 - change "probably" to "may".
L569 - 999.6398? The decimal places should match Figure 7.

Open Access This Peer Review File is licensed under a Creative Commons Attribution 4.0 International License, which permits use, sharing, adaptation, distribution and reproduction in any medium or format, as long as you give appropriate credit to the original author(s) and the source, provide a link to the Creative Commons license, and indicate if changes were made.

In cases where reviewers are anonymous, credit should be given to 'Anonymous Referee' and the source.
The images or other third party material in this Peer Review File are included in the article’s Creative Commons license, unless indicated otherwise in a credit line to the material. If material is not included in the article’s Creative Commons license and your intended use is not permitted by statutory regulation or exceeds the permitted use, you will need to obtain permission directly from the copyright holder.
To view a copy of this license, visit https://creativecommons.org/licenses/by/4.0/

Manuscript title: Breaking aromaticity in the anaerobic degradation pathway of phenanthrene comprises only ATP-independent type III aryl-CoA reductases

Manuscript ID: COMMSBIO-26-3141

Detailed Responses to Reviewers:

We acknowledge the efforts of the all Reviewers and their valuable comments that improved our manuscript. We would like to emphasize that all comments have been considered and notified in our manuscript. Please see our response and the accordance change in the entire manuscript (red color).

Response to the Reviewer #1:

The paper by Samak et al investigates the function of two members of the aryl-CoA reductase family, AprD and AprE that catalyze flavin-mediated reduction of intermediates in the anaerobic phenanthrene pathway. Importantly, the reactions function independently of ATP, yet are hypothesized to catalyze dearomatization of the benzene ring. This mechanism contrasts that of the naphthalene degradation pathway, where the benzene ring of tetrahydro-2-naphthoyl-CoA is reduced by an ATP-dependent type I aryl-CoA reductase.

Overall, the paper provides insight into the anaerobic degradation of PAH, revealing that dearomatization does not necessarily require ATP.

Major Comments:

Line 236 and line 225. Why is 1 mM NADH added to the reaction mixtures?

The reduction of 2-phenanthroyl-CoA to dihydro-2-phenanthroyl-CoA and the subsequent reduction to hexahydro-2-phenanthroyl-CoA required reduced methyl viologen as electron donor but the activity was higher with addition of NADH. The role of NADH in the reduction assay is probably to keep the flavins in a reduced state (52). The same was observed for the two reduction steps of hexahydro-2-phenanthroyl-CoA to decahydro-2-phenanthroyl-CoA. NADH is not electron donor here, because only 5% of the activity was retained when NADH was used as sole electron donor.

To clarify, the following sentence was added (now lines 495-496). “The function of NADH in the enzymatic assays with dithionite-reduced methyl viologen is probably to keep the flavins in a reduced state (42).”

Line 287. It is not clear how the authors were able to obtain pure quantities of hexahydro2-phenanthroyl-CoA, octahydro-2-phenanthroyl-CoA and decahydro-2-phenanthroyl-CoA.

The only substrate that could be synthesized and purified in the lab was 2-phenanthroyl-CoA. We were not able to purify the metabolites of hexahydro2-phenanthroyl-CoA, octahydro-2-phenanthroyl-CoA, and decahydro-2-phenanthroyl-CoA because elution peaks in the LC-MS chromatograms overlapped and the compounds were unstable and rearranged their electronic configuration when stored. Furthermore, the reactions were always incomplete remaining parts of the substrates.

To clarify, we added a sentence (now line 285-289). “Due to incomplete reactions, parts of the previous substrates 2-phenanthroyl-CoA, dihydro-2-phenanthroyl-CoA, and hexahydro-2-phenanthroyl-CoA always remained in the assays and purification of octahydro-2-phenanthroyl-CoA was not achieved. Nevertheless, the detection of single ion-traces allowed for clearly separating the compound peaks in the LC-MS.”

Figures 4-6 indicate that very little product was formed. How were these isolated from the starting material and quantified?

As explained in the previous question, we could not separate and purify the metabolites.

Line 278. It is not clear from the Methods and Materials section how the macroscopic kinetic parameters (kcat/specific activity and Km) were obtained for all three characterized enzymes. AprD, AprD and ApcA0. Data should be presented for the steady-state experiments. It is unclear how Km values in the nM range were obtained when the substrate concentrations varied from 1 -100 uM or 1 to 50 uM.

We found that we made a mistake while writing the methodology, the correct substrate concentrations are hexahydro-2-phenanthroyl-CoA **[6]** (1–200 nM), octahydro-2-phenanthroyl-CoA **[7]** (1–250 nM), and decahydro-2-phenanthroyl-CoA **[8]** (1-200 nM). We revised that section in the methodology carefully in lines 324-331 and also added the non-linear regression analysis in the supplement as **Fig. S5.**

Lines 295 to 511 are missing from the manuscript.

We are very sorry, we do not know how this mistake happen, probably while merging the files during the submission process.

Line 607. Please explain the unusually large errors associated with the FAD and FMN content. How were these SD values determined?

Values are reported as mean ± SD from three independent biological replicates, calculated from LC-MS quantification of extracted flavins normalized to enzyme concentration. Large deviations reflect variable cofactor occupancy, could also be due to the extraction method of flavins from the enzymes that was separately done in triplicates, besides the LC-MS ionization variability, but FMN and FAD were always detected. We added this explanation in the method section in lines 241-245. We also added in **table S2**, the independent values from each experiment, we also found that we overlooked the value of 0.7 ± 0.9 of the FAD content of AprE and it should be 0.7 ± 0.29, so, we corrected it in the **table 2**. The ± SD values were removed from the results section according to **Reviewer #2** comment.

Table 2. How was NADH 5% - maximal activity determined?

The activity obtained with NADH corresponded to approximately 5% of the maximal activity observed with dithionite-reduced methyl viologen as the electron donor, which was defined as 100% activity. We added this information under table 2, in lines 454-456.

Figure 1. If there is sequence similarity among ArpB, C, D, and E, it would make more sense, given the similarities in active-site geometry, that double bonds on the periphery of the rings would be reduced.

We added 3D alignment image using PyMol showing the fitting between the structure of the four enzymes of type III aryl-CoA reductases and 2-naphthoyl-CoA reductase to the supplementary file as image **Fig. S1**. We mentioned that image in the introduction section in line 120. As we discussed in the discussion, we consider the option of reduction of peripheral double bonds as alternative (now line 662-700).

Minor comments:

Line 47, remove "but water"

Corrected

Lines 54, 769, change to "monoaromatic"

Corrected in the whole manuscript

Line 60, change to "This constitutes a new degradation strategy that is distinct from the anaerobic degradation of…"

Corrected

Line 132: Rewrite the sentence beginning with "In this study…" It is not coherent in its present form.

The sentence was revised in lines 160-162.

Line 220. Remove "therefore" at the end of the sentence

Corrected in line 252.

Line 223 and elsewhere. Change "enzymatically accumulated" to "enzymatically formed"

Corrected

Line 224. Remove the second "reduction"

Corrected

Line 603. Small case "flavin mononucleotide"

Corrected in line 433.

Line 606. Small case "flavin"

Corrected in line 436.

Line 612 change to "cofactors"

Corrected in line 441.

Line 807 remove "kind of"

Corrected in line 642.

Line 812. Perhaps end the sentence with "ATP hydrolysis"

Corrected in lines 647.

Line 818. Split the sentence beginning with "Both types of" into two sentences.

Revised in lines 654-657.

Line 835: change to "to be asymmetric"

Corrected in line 671.

Line 883. Change to "It also catalyzes the"

Corrected in line 729.

Line 884. Remove the word "however"

Corrected in line 731.

Line 889. Change to "non-aromatic"

Corrected in line 736.

Response to Reviewer #2:

General comments

The study describes the last enzymatic steps in anaerobic bacterial phenanthrene degradation prior to ring-opening, comprising two subsequent two-electron reductions and an addition of water to the resulting cyclic diene system. Most important, it could be demonstrated that also the final two-electron reduction step from octahydro-2-phenanthroyl-CoA to decahydro-2-phenanthroyl-CoA yielding a fully dearomatized system is catalyzed by an ATP-independent type III aryl-CoA reductase. This is somewhat surprising, as related reactions in degradation pathways of benzene and naphthalene are catalyzed by ATP-dependent type I aryl-CoA reductases where ≥2 ATP are hydrolyzed per two-electron transfer to overcome the resonance energy of the aromatic system. Alternatively, ATP-independent type II aryl-CoA reductases are employed for the dearomatizing step, overcoming the resonance energy by energy input via an electron-bifurcating mechanism. In contrast, the "simpler" type III reductase-catalyzed reaction seem not to include any substantial energy input reaction mechanism to overcome the resonance energy.

Overall, the work is convincing. Unfortunately, lines L303 to L511 are missing in the manuscript (of the Materials and Methods part). The passages dealing with activation/resonance energies might be revised to some extent, as activation energies obviously differ (L822) in reducing aromatic 1-, 2- and 3-ring systems (see specific points). In addition, I found the low KM values (in the 30 to 150 nM range, Table 2) quite puzzling, as only substrate concentrations ≥1000 nM were tested to determine the kinetic parameters (L288). Maybe, you can provide corresponding kinetic plots and non-linear regression analysis in the supplement. Besides, I have several minor points that might be addressed to improve the manuscript (see below).

We agree and added **Fig. 9.** which shows the difference in ΔG for the reductions of benzoyl-CoA to cyclohexa-1,5-diene-1-carbonyl-CoA, and tetrahydro-2-naphthoyl-CoA (two rings) to the hexahydro-2-naphthoyl-CoA. These are compared with the ΔG of the reduction of the three aromatic ring system, dihydro-2-phenanthroyl-CoA to hexahydro-2-phenanthroyl-CoA, and octahydro-2-phenanthroyl-CoA to decahydro-2-phenanthroyl-CoA. From the image, it is very clear that the reduction of isomer **7p** of octahydro-2-phenanthroyl-CoA to decahydro-2-phenanthroyl-CoA is much easier than benzoyl-CoA to cyclohexa-1,5-diene-1-carbonyl-CoA, and the two aromatic ring tetrahydro-2-naphthoyl-CoA to the hexahydro-2-naphthoyl-CoA. That explains why no ATP hydrolysis is needed for the reduction of the peripheral rings of PAHs. To further clarify, we revised the discussion in lines 623-700, modifications are in red color.

We also calculated ΔGs for the reduction of each isomer of octahydro-2-phenanthroyl-CoA **[7]** to decahydro-2-phenanthroyl-CoA **[8]** and added them to **Table S3** in the supplementary file.

Regarding the low KM values, we found that we made a mistake while writing the methods section. The correct substrate concentrations are hexahydro-2-phenanthroyl-CoA **[6]** (1–200 nM), octahydro-2-phenanthroyl-CoA **[7]** (1–250 nM), and decahydro-2-phenanthroyl-CoA **[8]** (1-200 nM). We revised that section in the methodology carefully in lines 324-331 and also added the non-linear regression analysis in the supplement as **Fig. S5.** The apparent K_M value of AprD was typo mistake it should be 15.07 (15.1) instead of 150.7, we already corrected it in **table 2.**

Specific points

L31 "…two-electron reductions steps…" change "reductions" to "reduction" (see L62).

Corrected

L34 "Km" I would prefer "KM" with lowercase "M" (see Table 2).

Corrected in whole manuscript

L45 "pH value of 7.5" Ok, and for the other reactions?

The pH values of the other reactions were also 7.5 as indicated in lines 273, 293, and 311.

L54 "Anaerobic degradation of mono aromatic compounds and PAHs such as phenanthrene…" Abbreviation "PAH" not introduced (see L74).

Corrected

L56 "…that after phenanthrene is activated to a CoA-ester…" change "CoA-ester" to "CoA-thioester".

Corrected

L69, KEYWORDS: "culture TRIP 1" Maybe, you should change to "enrichment culture TRIP 1" (see L85).

Corrected

L78 "…stable PAHs and to brake the aromaticity…" Looks unusual to me. Maybe, change "brake" to "break".

Corrected in line 78.

L81 "…is well studied to a certain extent…" Well or to a certain extent? It is always studied to a certain extent. I would omit the "certain extent".

Corrected in line 100.

L82 "…biodegradation of phenanthrene and larger-PAHs [1]…", L84 "…degradation pathway of phenanthrene [1]…", L86 "…Trinidad and Tobago with phenanthrene [1]…" etc. Obviously, "[1]" refers to the compound phenanthrene (see also numbering of compounds in Fig. 1 and caption L152). But after introduction, you can just give the number and omit the compound name in the text. Otherwise we do not need the numbers. See also the numbering of the metabolite compounds (L92, 93…).

We appreciate this comment from the reviewer, but since we have a lot of compounds in our study, we believe using the compound name and the number will help the reader to follow-up and not to be lost with those all metabolites especially considering the figures. That was our experience from the last publications, most of the readers liked using the name and numbers.

L86 "…the Pitch Lake in Trinidad and Tobago…" I wonder whether there is only one ("the") pitch lake in Trinidad and Tobago. See ref. 10 (https://doi.org/10.1111/1462-2920.14335) = "at the Pitch Lake in La Brea, Trinidad".

Corrected to "Pitch Lake in La Brea, Trinidad" in line 105.

L94 "…the 2-naphthoate:CoA ligase (apaC) is located…" I think you mean the 2-phenanthroate:CoA ligase (of ref. 8).

Corrected in line 116.

L94 "…putative carboxylase gene clusters (apaA,B,F,G, I, and J)…" Well, the term gene cluster seems not to be well defined. But I would use here (= at least for the cluster of all apa genes) singular and not plural. See also L128 "constituting a gene cluster".

Corrected

L100 "genome of culture TRIP 1" As it is an enrichment culture but not a bacterial isolate (??), I would rather refer to the "metagenome". Or is it the MAG of Desulfatiglans TRIP_1? See also L114.

Corrected to "metagenome" in the whole manuscript.

L114 "In the genome of the anaerobic phenanthrene degrader TRIP 1"? Does "degrader" refer to an isolate?

It was corrected to "In the metagenome of the anaerobic phenanthrene enrichment culture TRIP 1,…" in lines 140-143.

L116 "…the neighborhood of the type III aryl-CoA reductase genes…" Well, the "neighborhood" is not essential. Are there genes for type I reductases in the genome/metagenome/MAG? And what about genes encoding ATP-independent type II aryl-CoA reductases? The latter seem also be relevant for sulfate-reducing bacteria (see Environ Microbiol 2019 Nov;21(11):4241-4252. doi: 10.1111/1462-2920.14784).

According to Kraiselburd et al., 2019, yes, there are genes in the TRIP 1 culture share 40 - 50% similarity with type I aryl-CoA reductase (5,6,7,8-tetrahydro-2-naphthoyl-CoA reductase) such as putative benzoyl-CoA reductase, N subunit, putative benzoyl-CoA reductase, O subunit, putative (R)-2-hydroxyglutaryl-CoA dehydratase activator, and Benzoyl-CoA reductase, Q subunit, but they are not in the neighborhood of the type III aryl-CoA reductase genes. Kraiselburd et al., 2019 who studied the metagenome of the TRIP 1 culture did not mention the presence of type II aryl-CoA reductases. So, the sentence was corrected in lines 1368– 143.

L117 to 121 "This is a very important problem because…" Well, that the Gibbs free energy change of the complete process (= multiple reactions) is negative is not sufficient. (And "delta G" is not an appropriate writing.) For each single reaction step (i.e., for each abiotic or enzymatically catalyzed reaction), the Gibbs free energy change must be negative = each step must be exergonic. Otherwise, the reaction(s) would not take place. Ok, obviously, the Gibbs free energy changes are "more or less in the same range for the different reduction steps of mono-aromatic compounds and PAHs". But is ref. 12 really appropriate for this statement? Does ref. 12 provide relevant values (of 1-, 2-, 3-ring systems) for the Gibbs free energy changes? And ref. 15 only deals with activation energy required for the dearomatization of the 1-ring system. This can obviously not simply be applied for other systems (see also your conclusion in L822). Are there any (theoretical) values available that describe activation energies for the different reduction steps? At least, the activation energies for the initial reduction steps of the 2- and 3-ring systems are lower than for the dearomatization of the 1-ring system. Consequently, type III reductases can catalyze the reactions, somehow "overcoming the resonance energy of the aromatic ring-system" (L96).

delta G is changed to ΔG in whole manuscript. Ref. 12 was removed from this sentence.

We added **Fig. 9.** which shows the difference in ΔG for the reductions of benzoyl-CoA to cyclohexa-1,5-diene-1-carbonyl-CoA, and the two ring tetrahydro-2-naphthoyl-CoA (one aromatic ring) to the hexahydro-2-naphthoyl-CoA, and compared them with ΔG of the reduction of the three aromatic ring system, dihydro-2-phenanthroyl-CoA to hexahydro-2-phenanthroyl-CoA, and octahydro-2-phenanthroyl-CoA to decahydro-2-phenanthroyl-CoA. From the image it is very clear that the reduction of isomer 7p of octahydro-2-phenanthroyl-CoA to decahydro-2-phenanthroyl-CoA is much easier than benzoyl-CoA to cyclohexa-1,5-diene-1-carbonyl-CoA, and the two aromatic ring tetrahydro-2-naphthoyl-CoA to the

hexahydro-2-naphthoyl-CoA. That's explain why no ATP hydrolysis needed. We revised the discussion in lines 623-700, modifications are in red color.
We also calculated ΔGs for the reduction of each isomer of octahydro-2-phenanthroyl-CoA **[7]** to decahydro-2-phenanthroyl-CoA **[8]** and added them to **Table S3** in the supplementary file. By the way, it is not true that each single step must be exergonic. Some exergonic reactions can pull endergonic reactions such as in e.g. syntrophic methanogenesis.

L124 "…PAHs with three (e.g. phenanthrene) or more rings can work if they do not possess…" Incomplete sentence. Specify "they".
The sentence was revised as following "Hence, it is unclear how type III aryl-CoA reductases can reduce the aromatic ring systems of larger PAHs with three (e.g. phenanthrene) or more rings without additional energy input from ATP-hydrolysis, if these larger PAHs do not use type I aryl-CoA reductases." In lines 149-152.

Fig. 1: Compound 3 "2- phenanthroyl-CoA" Remove spacebar. Generally, the "S" of the S-CoA is not in the same font size as the "O" or the "CoA". Why?

We thank the reviewer for this very good observation. We have corrected the image according to the reviewer's comment and also corrected the font and the size of the "S" of the S-CoA. Regarding how we write "CoA", CoA is the correct abbreviation according to the international organization of biochemical and enzyme Nomenclature IUBMB/UPAC.

Fig. 1, caption. "(a) Graphical presentation of the gene clusters encoding enzymes responsible for anaerobic phenanthrene activation (apaA-apaJ),…" In L94, you refer to the carboxylase genes as putative, indicating that the corresponding enzyme activity has not yet been established/identified. So, what is true? Responsible or putative responsible?
Corrected to "putatively responsible for" in Fig. 1 caption.

L265 "…the reaction was performed with or without 50 µM ATP." Why only 50 µM? Compared to the concentration of the electron donor components (added in the mM range) this is quite low.
We have tried different concentrations of the ATP (5 mM – 50 µM) with no effect, the problem with higher concentrations was it caused a lot of troubles to our LC-MS column, the ATP higher concentration was somehow precipitating in the column and caused blockage also to the

disolvation line (DL) of the MS, that's why we reduced the concentration and I wrote the lower concentration in case if another person repeated the experiment it does not cause column or DL blockage in the future. We revised the sentence in lines 301-302.

L294 "M. Braun…" Check formatting. Do you mean M. Braun Inertgas-Systeme GmbH?

Corrected to "MBraun Inertgas-Systeme GmbH, Garching, Germany" in lines 275 and 336-337.

L303 to L511 are missing in the manuscript!

We are very sorry, we do not know how this mistake happen, probably while merging the files during the submission process.

Results part: Do not repeat all the experimental details and what is already described in the Materials and Methods. For example, L577 "purified separately using a Strep-Tactin XT 4Flow gravity-flow column" or L645 "…the enzymatic reaction was stopped by adding a double volume of methanol. The methanol was evaporated, and the aqueous solution was freeze-dried to prepare a substrate stock of octahydro-2-phenanthroyl-CoA".

The repeated details were removed

Fig. 2: The gel image for ApcA in (b) looks somewhat distorted. I can hardly see the bands. Please check.

Another native gel image for ApcA was added to Fig. 2.

L605 etc. Do not simply repeat all the values (with SD) in the text that are nicely provided in Table 2.

The SD values were kept only in the table and removed from the text as the reviewer recommended.

L843 "If octahydro-2-phenanthroyl-CoA reductase AprD produces a non-aromatic isomer with conjugated double…". Yes, but then the AprD-catalyzed reaction would be challenged by (possibly) high activation energy to overcome the aromatic system.

There must be a little misunderstanding here. First, AprD is the hexahydro-2-phenanthroyl-CoA reductase.
And yes, the reviewer is right. But that's indeed the important point that the reduction of the aromatic rings is possible with type III reductases only. And we could demonstrate that in an earlier paper for 9,10-dihydro-2-phenanthroyl-CoA **[4c]** as well (Samak et al., *The ATP-independent dihydro-2-phenanthroyl-CoA reductase AprC catalyzes two consecutive two-electron reduction steps of dihydro-2-phenanthroyl-CoA to hexahydro-2-phenanthroyl-CoA in anaerobic phenanthrene degradation. Appl Environ Microbiol 0:e02248-25)*. This is discussed in the discussion in line 662-672.
So, we revised that part in the discussion in lines 660-700, modifications in red color.

Fig. 8: I wonder whether these calculations are also available for the naphthalene system. Could it explain the need for type I reductase employment and ATP-consumption in the last reduction step to yield the fully dearomatized system in the naphthalene pathway?
We added **Fig. 9.** which shows the difference in ΔG for the reductions of benzoyl-CoA to cyclohexa-1,5-diene-1-carbonyl-CoA, and the two aromatic ring tetrahydro-2-naphthoyl-CoA to the hexahydro-2-naphthoyl-CoA, and compared them with ΔG of the reduction of the three aromatic ring system, dihydro-2-phenanthroyl-CoA to hexahydro-2-phenanthroyl-CoA, and octahydro-2-phenanthroyl-CoA to decahydro-2-phenanthroyl-CoA. From the image it is very clear that the reduction of isomer **7p** of octahydro-2-phenanthroyl-CoA to decahydro-2-phenanthroyl-CoA is much easier than benzoyl-CoA to cyclohexa-1,5-diene-1-carbonyl-CoA, and the two ring tetrahydro-2-naphthoyl-CoA (one aromatic ring) to the hexahydro-2-naphthoyl-CoA. That's explain why no ATP hydrolysis needed. We revised the discussion in lines 623-700, modifications are in red color.
We also calculated ΔGs for the reduction of each isomer of octahydro-2-phenanthroyl-CoA **[7]** to decahydro-2-phenanthroyl-CoA **[8]** and added them to **Table S3** in the supplementary file.

Response to Reviewer #3:

This is a very nice study that successfully illuminates some new biochemistry in the anaerobic degradation of hydrocarbons. Enzyme candidates have been identified, heterologously expressed and purified, and a pathway for phenanthrene degradation reconstituted in vitro. The paper is very well written overall with few typographical errors although the figures could be clearer. The data appear to be in order and the

methods are described with sufficient clarity to permit reproduction. Some minor comments:

Abstract Line 27 – why are these enzymes specifically described as 'ATP-independent'; most enzymes are?

Not all of the enzymes involved in PAHs reduction are ATP-independent, for example, the reduction of 5,6,7,8-tetrahydro-2-naphthoyl-CoA to hexahydro-2-naphthoyl-CoA is ATP-dependent. So far, the reduction of a remaining benzene ring was only known to be ATP-dependent, probably because of the high activation energy.

This is discussed in line 80-99.

Line 78 'break'

Corrected in line 78.

Fig 1 – harmonise size of S atom labels in these structures – not clear why they are smaller than others

We have corrected the image according to the reviewer's comment and corrected the font and the size of the "S" of the S-CoA.

Methods – min, not minutes; Michaelis 'K' is italic

Corrected in the whole manuscript.

Line 598 – use 'OYE' early in the manuscript – this is an accepted abbreviation and can then be used throughout

Corrected in the whole manuscript.

Line 612 – change 'optically active' as this may inadvertently suggest chirality and the preponderance of one enantiomer

Revised in line 441.

Figure 4 Page 22 – legibility of text and structures is an issue here – please reconsider format; also Figure 5

Text and structures were revised in Figures 4, 5, and 6.

Line 742 remove 'is real'

Removed

Figure 8 – need to rethink this; again for legibility and the proper representation of chemical structures.

Figure 8 was revised according to the reviewer's comment.

Response to Reviewer #4:

It's been an exciting year for the field of anaerobic hydrocarbon biodegradation. Several new papers and preprints have been published on the characterization of associated enzymes and metabolic reactions, especially for PAHs. Drs. Samak, Meckenstock, and their team have published a series of papers on the characterization, heterologous expression, and activity of enzymes involved in phenanthrene biodegradation under sulfate-reducing conditions, specifically 2-phenanthroate:CoA ligase (10.1128/aem.01296-24), phenanthroyl-CoA reductase (10.1128/aem.00166-25), and dihydro-2-phenthanthroyl-CoA reductase (10.1128/aem.02248-25). This fourth paper continues these efforts and describes three new enzymes that participate in ring reduction (AprD, AprE) and hydrolysis steps (ApcA) of anaerobic phenanthrene degradation by a Desulfatiglans-affiliated bacterium in the sulfate-reducing enrichment culture TRIP 1.

Comments:

Prior to reading this latest paper, I was aware that the Meckenstock lab had enriched for a phenanthrene-degrading enrichment culture (Himmelberg et al., 2018; doi:10.1111/1462-2920.14335) but was not well versed in their more recent enzyme characterization efforts. Figure 1 and Lines 84-125 provide a helpful overview of the aforementioned papers and their major discoveries. However, I found that some concepts central to this paper were glossed over. For example, what exactly are type III aryl-CoA reductases, when were they first described, and how do they differ from type I and type II aryl-CoA reductases? What is the old yellow enzyme family? Are all old yellow enzymes type III aryl-CoA reductases? It was only after I carefully read through Samak et al.'s previous papers that I found answers to some but not all of these questions. While each paper builds on the last, which supports continuity, this approach risks leaving key concepts insufficiently defined for readers who are not already familiar with the series. The authors should be mindful of this, and be sure to introduce all relevant concepts, organisms, and enzymes in sufficient detail when they first appear in each manuscript.

We revised the introduction section and responded to the comments in lines 80-152.

The experiments and data presented in this study are technically sound, and the work is overall very impressive. One Materials and Methods section (starting on line 291) appears to be missing a substantial amount of text (perhaps due to a copy/paste error) and should be corrected.

We are very sorry, we do not know how this mistake happen, probably while merging the files during the submission process.

I do have a few questions regarding this study's enzyme activity assays. The CoA-ester substrates used for enzyme activity assays were synthesized enzymatically. Based on the t = 0 min data presented in Figures 4 and 5, it appears that the target CoA-ester substrates were not (or could not be) purified prior to use (i.e., residual 2-phenanthroyl-CoA, 7,8-dihydro-2-phenanthroyl-CoA, etc., remain present). Please confirm whether this is the case. If so, could these residual compounds have influenced the apparent rates of AprD, AprE, and ApcA? Additionally, what reference standard(s) or LC-MS calibration approaches were used for quantification of each synthesized CoA-ester?

The only substrate that could be synthesized and purified in the lab was 2-phenanthroyl-CoA (our reference standard). We were not able to purify the metabolites of hexahydro2-phenanthroyl-CoA, octahydro-2-phenanthroyl-CoA, and decahydro-2-phenanthroyl-CoA because elution peaks in the LC-MS chromatograms overlapped and the compounds were unstable and rearranged their electronic configuration when stored. Furthermore, the reactions were always incomplete remaining parts of the substrates.
To clarify, we added a sentence (now line 285-289). "Due to incomplete reactions, parts of the previous substrates 2-phenanthroyl-CoA, dihydro-2-phenanthroyl-CoA, and hexahydro-2-phenanthroyl-CoA always remained in the assays and purification of octahydro-2-phenanthroyl-CoA was not achieved. Nevertheless, the detection of single ion-traces allowed for clearly separating the compound peaks in the LC-MS."
We agree that the residual compounds present in the enzymatic assays might have affected the apparent rates of AprD, AprE, and ApcA which is now discussed in line 470-471.

Well done, authors! Some specific line comments below.

L84: "In this study, we uncover the upper anaerobic degradation pathway of phenanthrene …" This is somewhatt misleading. This paper only describes three steps in this pathway.

Corrected to "we continue our investigation to uncover" in line 103.

L87-88: "The dominant organism of culture TRIP 1 belongs to the genus Desulfatiglans within the family Desulfobacteraceae". How many organisms are present in TRIP 1, and what proportion of the community is comprised of Desulfatiglans? Himmelberg et al.

(2018; doi:10.1111/1462-2920.14335) describes four organisms based on metagenomic sequencing and MAG assembly (Desulfobacteraceae/Desulfatiglans, Geobacter, Paludibacter, and a Spirochaete.) Has this community structure changed since ~2018, and how is it being monitored? As an aside, I checked the NCBI entry for the TRIP 1 metagenome and assembly data (PRJEB27924) and found that the Desulfatiglans genome assembly was suppressed (annotation fails MAG completeness check; see https://www.ncbi.nlm.nih.gov/datasets/genome/GCA_900498135.1/). Please review. Has a more recent Desulfatiglans genome been sequenced and assembled?

We have another article published by Kraiselburd, et al., 2019 after the investigation done by Himmelberg et al. (2018), which shows that the relative abundance of genomes reconstructed from the TRIP metagenome indicate *Desulfobacteraceae, Desulfatiglans* (60.4 %), *Porphyromonadaceae, Paludibacter* (19.6%), *Desulfobulbaceae* (6.8%), *Spirochaetaceae, Spirochaete* (3.4%), *Desulfomicrobiaceae, Desulfomicrobium* (1.8%), and *Desulfarculaceae,* Desulfarculus (1.3), and 6.7% of other operational taxonomic units. We added that to the introduction in lines 106-109.

The community structure was not changed since that time, one reason could be the very slow growth rate of the TRIP 1 culture. The community composition was monitored by 16S rRNA gene amplicon sequencing, that was performed last time in 2022. No more genome assembly has been performed to be added to the database.

L89-107: The authors should clarify that the phenanthrene degradation pathway overviewed in this paragraph is specific to Desulfatiglans in TRIP 1 and is not necessarily a universal phenanthrene degradation pathway. We do not yet know the full breadth of microbes and biochemical mechanisms capable of catalyzing PAH degradation. Anaerobic benzene biodegradation, for example, can be initiated by several activation mechnanisms including but limited to carboxylation.

The sentence “The initial steps of the anaerobic degradation pathway of phenanthrene [1], using enzymes encoded by the genes of the genus *Desulfatiglans* in enrichment culture TRIP 1,….” was added to the paragraph in lines 110-111.

L104: What is “two times two-electron reduction”?

Corrected to “two consecutive two-electron reduction” in line 128.

L112-113: Please consider introducing Azoarcus, Deltaproteobacteria N47 and strain NaphS2 as model organisms used to study anaerobic benzene and PAH biodegradation, as well as all three types of aryl-CoA reductase enzyme families, to make the manuscript more accessible to readers who are not subject-matter experts.

We added all these information to the introduction section in lines 80-152.

L129: What does thn stand for? The authors may also wish to define the abbreviations for apr and apc in the main text, as they are easy to miss in Figure 1.
thn stands for tetrahydro-2-naphthoyl-CoA gene cluster, it is revised in the manuscript in line 157.
the abbreviations for apa, apr and apc were defined in the text in line 115, 120, and 156.

Figure 1: The authors may wish to consider adding citations to specific papers under each characterized reaction step so that readers can locate relevant studies more easily. In the figure caption, please explain why is there a red X through 9,10-dihydro-2-phenanthroyl-CoA (4c).
Citations were added to the caption of Figure 1. The red X was explained in the caption.

L195: Why were these volumes chosen? Please provide additional detail.
These volumes were recommended by IBA-lifesciences instructions. We added this info in line 224-225.

L214: This is a very old reference (from 1963). Is there a more recent protocol that can be cited, particularly one that reflects updates to the original procedure?

The reference is updated to *(Zuo S, Lundahl P. 2000. A micro-Bradford membrane protein assay. Anal biochem.)* In line 228.

L580: Any insights into the identity of this impurity?
The additional band observed at approximately 98 kDa likely represents a co-purifying endogenous *E. coli* protein retained during affinity purification. However, without mass spectrometry analysis, its identity remains uncertain.

L583: Should be ApcA (capital A).
Corrected in line 413.

L592: Should be kDa (lowercase k). Please review the text carefully for similar formatting errors.
Corrected in whole manuscript.

Table 2: Consider specifying the number of replicates performed per assay and what the error bars represent.
We added the non-linear regression analysis for the enzymatic assays and added what error bar represent in the supplement as **Fig. S5 & Table S2.**

L657-660: I was surprised to read that AprD and AprE do not appear to be oxygen sensitive. How does this compare to other characterized "old yellow" and type III aryl-CoA reductases?

Yes, the enzymes are not oxygen sensitive and we can simply produce and purify them under oxygen condition. However, since these enzymes have flavin and the reduction assay need that these flavins to be reduced that's why we need to flush the enzymes with nitrogen then put them inside the anaerobic chamber to perform the enzymatic assay.

Type III aryl-CoA reductases catalyze the reduction of 2-naphthoyl-CoA to 5,6-dihydro-2-naphthoyl-CoA and the subsequent reduction of the latter to 5,6,7,8-tetrahydro-2-naphthoyl-CoA discovered in sulfate-reducing Deltaproteobacteria N47 and strain NaphS2 are also not oxygen sensitive. But OYEs are not oxygen sensitive in general so, we added the sentence "However, not all OYEs are insensitive to oxygen (41)." In line 490.

We revised that in the introduction in lines 80-102 and 131-135 and in the results section in lines 485-490.

L665-666: Are these data presented anywhere? It might be worthwhile to include them as Supplemental Information, including electron donor concentrations. Negative results can be informative for other research groups working on enzyme characterization.

The concentrations of different electron donors used in this study is presented in the method section in lines 298-301.

L764-766: "…which is performed by either a fumarate addition to methyl groups or a carboxylation of the aromatic ring in the case of non-substituted compounds such as benzene and PAHs." Fumarate addition and carboxylation are not the only known biochemical mechanisms of anaerobic hydrocarbon activation.

Corrected in line 595-596.

L800-802: Now that several type III aryl-CoA reductases have been characterized, it would be worthwhile to construct a phylogenetic tree of this enzyme family. Consider including uncharacterized homologs from hydrocarbon-degrading isolates and enrichment cultures beyond NaphS2 and N47. This would be very helpful for understanding the substrate diversity and evolutionary diversity of type III aryl-CoA reductases. Bootstrap support values should be included.

We thank the reviewer for this valuable suggestion. While we agree that a comprehensive phylogenetic analysis would be informative, such an extensive study is beyond the scope of

our current article which mainly focus on the biochemical characterization of the three enzymes, but we will consider that nice suggestion for our next article after we finish the crystallization experiments of these enzymes.

L829: "… is supported by previous results …" Please provide an appropriate citation.
Citation added in line 665.

L867-875: Consider preparing a phylogenetic tree for ApcA and representative enzymes within the crotonase superfamily. Does ApcA form a distinct clade or does it cluster with known enzyme groups? The reported 42-60% gene sequence identity is very high - did you mean amino acid identity?
Yes, we mean the amino acid identity, we revised that in the discussion in lines 718-719. We also thank the reviewer for this valuable suggestion. Since we are planning to have another article after we finish the crystallization experiments of the ApcA enzyme, we will consider this suggestion for our next article.

We hope this amendment will take your consideration. We again appreciate these thoughtful and valuable comments.

Manuscript title: Breaking aromaticity in the anaerobic degradation pathway of phenanthrene comprises only ATP-independent type III aryl-CoA reductases

Manuscript ID: COMMSBIO-26-3141B

Detailed Responses to Reviewers:

We acknowledge the efforts of the Reviewers to read the manuscript again with such care. Their valuable comments improved our manuscript. We would like to emphasize that all comments have been considered and notified in our manuscript. Please see our response and the accordant changes in the entire manuscript (red color).

We want to emphasize that we edited the manuscript according to the editor's request to follow the journal formatting style, therefore, we have removed the "Importance" section and the subheadings of the "discussion" section from the manuscript, and we also moved the method section to the end of the manuscript. We added "Statistics and reproducibility", "Reporting summary", "Data availability", and "Author contributions" sections at the end of the manuscript.

Response to Reviewer #2:

I do appreciate that most of my comments have been addressed appropriately in the revision of the manuscript. However, there are still things to do. It is good that you have corrected several (serious) typos (KM and SD values, concentration units of substrates used in assays). But you have to correct them throughout (see L34). And sometimes, the revision did not improve the manuscript (e.g. L149, see comment below). Moreover, to be as consistent and correct as possible in a manuscript is not just for fun. It is important for clearness and will help to prevent propagation of errors in future studies. You should also not state in the rebuttal that something was corrected when it is not (e.g. see comment below on L116).

We appreciate the reviewer's careful revision of our manuscript and apologize for the unintentional oversight.

L34 "12.7 and 6.3 nmol min-1 mg-1 and KM values of 150.7 and 63.9 nM, respectively." In the rebuttal, you indicated a typo ("The apparent KM value of AprD was typo mistake it should be 15.07 (15.1) instead of 150.7,…"). And in Table 2 it is corrected. But you have to correct it throughout!

We have corrected the KM value according to Table 2 (which became now Table 1) in line 34 and we also revised it throughout the manuscript to make sure that the values are correct.

L92 "old-yellow enzyme family (OYE)" change to "old-yellow enzyme (OYE) family" (see L40, L58 etc.).

We have changed "old-yellow enzyme family (OYE)" to "old-yellow enzyme (OYE) family" throughout the manuscript.

L116 "The gene encoding the 2-naphthoate:CoA ligase (apaC) (25) is located in the vicinity of the putative carboxylase gene cluster…" In the rebuttal, you stated that it was corrected. But it is still "2-naphthoate:CoA ligase", although the context and ref. 25 indicate that it should be the "2-phenanthroate: CoA ligase". See L115 (ApaC = phenanthroate:CoA ligase).

Sorry for this mistake, we have revised "2-naphthoate:CoA ligase" to "2-phenanthroate: CoA ligase" in line 102.

L144 "This is a very important problem…" I think the passage is ok. Just a small reply on your rebuttal comment "Some exergonic reactions can pull endergonic reactions such as in e.g. syntrophic methanogenesis." Yes, the effective concentration (activity) of hydrogen is kept very low in this synthrophy, resulting in the "pull" from endergonic to exergonic. But, of course, endergonic refers here to standard conditions and also to a wide range of other hydrogen activity values. At very low hydrogen activity (at real conditions and due to the very efficient hydrogen consumption in the synthrophic system), however, the reaction is exergonic = it can take place. Standard conditions or other reference conditions (e.g. at neutral pH) are useful to compare different reaction steps in a pathway and to identify thermodynamic bottlenecks. But we have to keep in mind that in the real world, the Gibbs free energy change (of each single reaction) depends on the real conditions (temperature, activity values of substrates and products). So, when giving values for the Gibbs free energy change, you should indicate the conditions (e.g. standard conditions corrected for pH = 7). See also L652 and Fig. 9.

To clarify, we changed the ΔG in line 131 to $\Delta G^{0'}$ to make sure that we talk about standard conditions and the pH 7 was added in line 134. We also changed to "($\Delta G^{0'} \approx$ -60 kJ mol^{-1} at physiological conditions in the cell)" in line 431 and the conditions were added in the legend of Fig. 8 (previously Fig. 9) in line 473.

L149 "Hence, it is unclear how type III aryl-CoA reductases can reduce the aromatic ring systems of larger PAHs with three (e.g. phenanthrene) or more rings without additional energy input from ATP-hydrolysis, if these larger PAHs do not use type I aryl-CoA reductases. I am sorry, but this is not better than before. PAHs "do not use" something. The original sentence was: "Hence, it is unclear how the reduction of the aromatic ring systems of larger PAHs with three (e.g. phenanthrene) or more rings can work if they do not possess type I aryl-CoA reductases." I think the "they" should refer to the degrading system = the microorganisms. They can possess or lack the relevant enzymes.

Very true, thanks for the clarification. The sentence is revised as following "Hence, it is unclear how anaerobic microorganisms can use type III aryl-CoA reductases to reduce the aromatic ring systems of larger PAHs with three (e.g. phenanthrene) or more rings without additional energy input from ATP-hydrolysis, instead of using type I aryl-CoA reductases." In lines 136-139.

L248 "Enzymatic synthesis of CoA-ester substrates"
L306 "phenanthroyl-CoA ester [8]"
L307 "decahydro-2-phenanthroyl-CoA ester"
L310 "decahydro-2-phenanthroyl-CoA ester"
L315 "hydroxydodecahydro-2-phenanthroyl-CoA ester"
L442 "of 2-phenanthroyl-CoA ester"
Change "ester" to "thioester" (see L56) or omit "ester" where it is not needed (e.g. L315).

"ester" was changed to "thioester" in lines 594, 617, 625, 628, 637, 643, 652, 653, 656, 661, and 689**.**

L330 "Vmax" Use consistent writing throughout (see L325 = italic and subscript).

Revised in line 677 and in the whole manuscript.

L440 "…showed an absorption at 280 nm, …" Maybe, change to "…showed only an absorption peak at 280 nm, …". Of course, also the flavin-containing enzymes show substantial absorption at 280 nm, but ApcA lacks the peaks around 375 and 450 nm.

Corrected in line 211.

Fig. 6, L558 "β-hydroxydodecahydro-2-phenanthroyl-CoA [9]". Sorry for not commenting in my first review. But looking at Fig. 6 again, I am not sure with the

structure of the ApcA reaction product = compound [9]. In Fig. 1, you indicate two isomers for [9], but here only one. See also section title ("…adds a water molecule to one of the double bonds…"). Could LC-MS confirm the isomer depicted in Fig. 6? Or is it possible that the other or that both isomers were formed?

We thank the reviewer for this comment. Yes, it is possible that one of the two isomers is produced, but we do not know exactly which one because the LC-MS and the Q-TOF/LC-MS analysis cannot detect the position of the water molecule addition. To clarify, we changed the text in lines 320-321 to "…to one of two possible isomers of the β-hydroxydodecahydro-2-phenanthroyl-CoA [9]…" we also added the sentence "…, producing one of two possible isomers of β-hydroxydodecahydro-2-phenanthroyl-CoA [9].." to the legend of Fig. 6 in lines 333-334 and added a sentence to make clear that these are only exemplary structures: "The position of the double bonds in the structures of decahydro-2-phenanthroyl-CoA **[8]** and β-hydroxydodecahydro-2-phenanthroyl-CoA [9] are shown exemplarily since the exact isomeric structures are not known" in line 335-337. We also added the second possible isomer to Fig. 6.

L670 "SP3 hybridized" change to "sp3 hybridized" with the "3" as superscript.

Corrected in line 450.

L648, L652, L684, L685, Fig. 8, L692 "kJ/mol" I would prefer "kJ mol^{-1}" **with "-1" as superscript. See L678.**

Corrected in the whole manuscript and updated in Fig. 9 (previously Fig. 8).

Fig. 9, L695 "Gibbs free energies" I would prefer "Gibbs free energy changes". And, as always, you have to give the units (I guess the values are in kJ mol-1).

Fig.9 now became Fig. 8 and "Gibbs free energies" changed to "Gibbs free energy changes" in line 473, and the unit is added in line 474.

Response to Reviewer #4:

It was a pleasure to revisit this manuscript. My questions and comments were appropriately addressed by the authors; thank you. I look forward to learning more about the phylogeny of type III aryl-CoA reductase and ApcA in your forthcoming manuscripts.

We appreciate the reviewer's careful revision of our manuscript.

A few minor corrections:

L84 - It's odd to start citing your chemical structures from [13] (benzoyl-CoA), which is first shown in Figure 9. Consider removing [13] here to avoid a lot of reformatting.

[13] was removed from the line.

L109 - 1.3 is missing a %; Desulfarculus should be italicized.

Corrected in line 95.

L121; 141 - are you referring to amino acid or nucleotide sequence identities? This should always be specified.

We have revised that section as you can see in lines 107, 127, and 143.

L164 - apcA should be lowercase and italicized.

Corrected in line 151.

L243 - SD should be written out in full.

Corrected in line 589.

L330 - Vmax formatting should match the rest of the main text.

Corrected in the whole manuscript.

L496; 685 - change "probably" to "may".

Corrected in lines 269 and 465.

L569 - 999.6398? The decimal places should match Figure 7.

The decimal places in this section and the figure legend were written as indicated in the figure in lines 341-365.

We hope this amendment will take your consideration. We again appreciate these thoughtful and valuable comments.
